# Self-Assembly of Surfactants in Ionic Liquids: Mechanisms, Applications and Recent Developments

**DOI:** 10.3390/molecules31203767

**Published:** 2026-10-20

**Authors:** Yue Zhao, Xingwang Li, Yunze Li, Shulin Liu, Junshuang Wu

**Affiliations:** 1 Key Laboratory of Ecological Restoration of Regional Contaminated Environment, Ministry of Education, College of Environment, Shenyang University, Shenyang 110044, China; 2 College of Science, Shenyang University of Chemical Technology, 11th Street, Shenyang Economic and Technological Development Zone, Shenyang 110142, China; 3 Chemistry and Chemical Engineering Institute, Qiqihar University, Qiqihar 161006, China

**Keywords:** ionic liquids, surfactants, self-assembly, solvophobic effect, lyotropic liquid crystals, structure–property–performance relationships

## Abstract

Surfactant self-assembly in ionic liquids (ILs) has attracted increasing attention because ILs provide highly tunable ionic environments that differ fundamentally from conventional aqueous media. Unlike water-driven aggregation dominated by the hydrophobic effect, self-assembly in ILs is governed by the cooperative interplay of solvophobic interactions, electrostatic screening, hydrogen-bond networks, specific ion effects, and solvent nanostructuring. Although substantial progress has been achieved during the past decade, a unified mechanistic understanding that links IL structure to aggregate morphology and functional performance remains lacking, and the literature is fragmented across system classes that are rarely compared under matched experimental conditions. This review presents a mechanism-oriented synthesis of surfactant self-assembly in IL media. The review is organized around an explicitly defined classification of IL-amphiphile systems, distinguishing neat IL media, IL–water mixtures, surface-active ionic liquids (SAILs), ILs used as cosurfactants, and conventional surfactants dissolved in ILs, and it states the literature-search strategy and eligibility criteria used to assemble the evidence base. The fundamental driving forces responsible for aggregation are then analyzed critically, with emphasis on how solvophobic interactions, electrostatic screening, hydrogen bonding, and packing-parameter modulation jointly determine aggregate formation and structural evolution, and on the quantitative thermodynamic and structural parameters, namely the Gibbs energy, enthalpy, and entropy of micellization, the critical micelle concentration (CMC), and the aggregation number, that make these contributions comparable between systems. Representative self-assembled architectures, including micelles, reverse micelles, lyotropic liquid crystals, microemulsions, and worm-like micelles, are discussed together with the resolution limits, artifacts, and applicable concentration ranges of the characterization techniques and simulation approaches used to resolve them. Applications in nanomaterial synthesis, electrochemical energy storage, drug delivery, enhanced oil recovery, and green separation technologies are reorganized around explicit structure–property–performance relationships, and claims that currently rest on schematic or correlative evidence are identified as such. Finally, a critical roadmap is presented that addresses reproducibility, standardized reporting, viscosity and transport limitations, impurity and water sensitivity, scale-up, cost, toxicity, recyclability, and quantitative predictive design, including realistic opportunities for machine-learning-assisted modeling. By integrating recent advances from molecular mechanisms to practical applications, this review provides a unified framework for understanding surfactant self-assembly in ionic liquids and offers guidance for the rational design of next-generation soft functional materials.

## 1. Introduction

Surfactants are a typical class of amphiphilic molecules, consisting of a hydrophilic headgroup and a hydrophobic tail chain, which are capable of orienting themselves at interfaces and significantly reducing the surface tension or interfacial tension of a system [1]. Under appropriate conditions, surfactant molecules can spontaneously aggregate to form various ordered structures, such as micelles [2], vesicles [3], microemulsions [4] and lyotropic liquid crystals [5]. This self-assembly behavior is not only one of the core issues in colloid and interface science, but also plays a significant role in fields such as catalysis [6], drug delivery [7], nanomaterial synthesis [8] and energy conversion [9]. Historically, research has focused on aqueous systems, where the hydrophobic effect dominates. The limitations of water-based media, namely poor thermal stability, narrow electrochemical windows, and low salt tolerance, have motivated the exploration of non-aqueous solvents such as ionic liquids [10]. Demand for operation under high-temperature, high-salt, and strictly non-aqueous conditions has reinforced this shift, because conventional aqueous formulations lose structural integrity or fail outright under such conditions.

Ionic liquids (ILs), as ionic substances that remain liquid near room temperature, are regarded as a new generation of functional solvents [11] due to their low vapor pressure [12], wide electrochemical window [13], high thermal stability, and structural designability [14]. Unlike traditional molecular solvents, ionic liquids consist of anions and cations, with strong Coulombic interactions [15], hydrogen-bond networks [16], and nanoscale structural heterogeneity [17] present within the system. This highly ordered and tunable solvent environment provides a new dimension for modulating the aggregation behavior of amphiphilic molecules [18]. Studies have shown that in ionic liquids and their mixtures, surfactants can still form a variety of self-assembled structures, although the driving mechanisms differ significantly from those in aqueous systems [19]. In addition to the traditional solvophobic effect [20], high ionic strength [21], specific intermolecular interactions [22], and the inherent nanostructure of ionic liquids [23] may all exert a decisive influence on the critical micelle concentration(CMC), aggregation morphology, and structural stability.

In recent years, research on surfactant–ionic liquid systems has developed rapidly, covering the modulation of aggregation behavior, the mechanisms of mixed micelle formation, interfacial adsorption properties, and applications in fields such as nanomaterial preparation [24], biomedicine [25], energy storage [26] and enhanced oil recovery [27]. Micelles [28], vesicles [29] and even liquid crystal phases [30] have been observed in a range of imidazolium-based ILs, and their use in the template synthesis of nanomaterials [31] and in enhanced oil recovery [32] has been explored. Three gaps nonetheless motivate the present review. First, few systematic accounts exist of how self-assembly behavior varies across IL classes, for example between protic and aprotic systems and between long-chain and short-chain cation structures. Second, no unified framework yet describes how electrostatic interactions [33], hydrogen bonding [34], van der Waals forces [35] and solvation effects [36] cooperate or compete under the strongly correlated conditions of an IL medium, and the relative weight of these contributions is rarely established for a given system. Third, the environmental and toxicological credentials of IL-based systems remain contested [37], and the evidence base for their large-scale use is uneven. Consequently, a comprehensive review and critical analysis of the self-assembly mechanisms of surfactants in ionic liquids, and of the application claims built on them, is necessary.

Against this background, this work provides a comprehensive review of the self-assembly behavior of surfactants in ionic liquids and of the mechanisms that modulate it. It begins by introducing the basic structural characteristics and physicochemical properties of surfactants and ionic liquids; subsequently, the driving forces behind self-assembly are analyzed from the perspectives of solvophobic effects, electrostatic shielding, hydrogen-bond networks and molecular synergy; further, recent application progress in the fields of nanomaterials, biomedicine, separation science and green chemistry is summarized; finally, the challenges facing current research and future development trends are discussed. This paper aims to provide a systematic mechanistic overview for a deeper understanding of the self-assembly behavior of amphiphilic molecules in non-aqueous and high-ionic-strength environments, and to offer guidance for the design of novel functional soft-matter systems and green processes.

### 1.1. Objective and Novelty of This Review

Several reviews have examined ionic liquids as media for amphiphile self-assembly, but they tend to be organized around a single system type or a single application domain. Moshikur et al. reviewed surface-active ionic liquid-assisted self-assembly specifically for drug delivery [38]; Ban et al. examined co-assembly routes to mesoporous nanospheres [39]; and Warr and Atkin analyzed solvophobicity as a driving force in neoteric and nanostructured solvents [40]. Where broader overviews exist, they describe the phenomenology of solvophobicity and nanostructure without translating the underlying interactions into quantitative design rules, and they rarely separate the physical behavior of neat IL media from that of IL–water mixtures or of surface-active ionic liquids.

This review differs from that literature in four respects. First, it defines the system classes explicitly and keeps them separate throughout, so that a mechanistic statement established for neat IL media is not silently extended to IL–water mixtures, to SAILs, or to systems in which the IL acts only as a cosurfactant (Section 1.2). Second, it makes the evidence base transparent by reporting the literature-search strategy and the eligibility criteria used to assemble it (Section 1.3). Third, it treats the mechanism quantitatively, tabulating the thermodynamic and structural parameters that can be extracted from experiments and stating the conditions under which the classical models fail (Section 3.7, Table 1). Fourth, it evaluates application claims against the strength of the supporting evidence, separating effects that have been resolved to specific self-assembled structures from effects that are at present correlative (Section 5, Table 2).

### 1.2. Definition of the System Classes Covered

Terminology in this field is used inconsistently, and comparisons are frequently drawn between physically distinct systems. Five classes are distinguished throughout this review and are summarized in Table 3.

Neat ionic liquid media. The continuous phase is an IL, with no deliberate addition of water or molecular cosolvent. Residual water is unavoidable at some level and is treated as a variable in Section 2.5. Aggregation in these systems is driven principally by solvophobic partitioning of the surfactant tail into the non-polar domains of the IL [40].

Ionic liquid–water mixtures. Water is present as a major component and usually forms the dominant continuous phase, so that the IL acts as a cosolvent, electrolyte, or hydrotrope. The hydrophobic effect remains the leading driving force, modified by ion-specific effects and by the disruption of water structure [41].

Surface-active ionic liquids. The amphiphile itself is an ionic liquid, most often through a long alkyl chain attached to the cation, and the amphiphile therefore shares its chemical architecture with the medium. This structural matching changes both the solvation of the headgroup and the way in which the aggregate interface is organized [42]. Systematic variation in the cation, anion, and chain length within this class has produced some of the clearest structure–property correlations available [43,44].

Ionic liquids used as cosurfactants. The IL is added to a surfactant-oil–water formulation at concentrations well above the trace level and participates directly in the interfacial film, altering the curvature and the interfacial tension [45,46].

Conventional surfactants dissolved in ionic liquids. The amphiphile is a conventional cationic, anionic, nonionic, or zwitterionic surfactant and the IL is the medium. Studies in this class illustrate how the aggregation threshold of a well-characterized surfactant responds to a change in solvent [47] and how that response differs between IL families and between IL–water mixtures [45,46].

These classes are not mutually exclusive, and the boundary between them is set by composition rather than by chemical identity. A system described as anhydrous becomes a member of the second class once the water concentration approaches or exceeds the surfactant concentration, and a formulation that contains an IL above its solubility limit in water belongs to the second class rather than to the first. Where a study does not report the composition needed to assign a class unambiguously, this is stated in Table 4, and the corresponding result is treated as provisional.

### 1.3. Literature Search and Selection Strategy

This review is presented as a critical synthesis rather than as a formal comprehensive review, but the search procedure is reported so that readers can judge the coverage and reproduce the selection. Records were retrieved from Web of Science, Scopus, and Google Scholar, supplemented by backward and forward citation tracking from the central primary studies. The search window was January 2010 to March 2026; earlier work is cited only where it defines a concept or a benchmark that later studies continue to use. Search strings combined a solvent term, an amphiphile term, and a behavior term, for example (“ionic liquid*” OR “molten salt*” OR “surface-active ionic liquid*”) AND (surfactant* OR amphiphile* OR “block copolymer*”) AND (self-assembl* OR micell* OR aggregat* OR “liquid crystal*” OR microemulsion* OR “worm-like micelle*”). The strategy, the eligibility criteria, and the fields extracted from each study are summarized in Table 5.

Records were eligible if they reported experimental or computational evidence on amphiphile self-assembly in a medium containing an ionic liquid, or if they reported physicochemical data for such a system, including the critical micelle concentration, the aggregation number, thermodynamic parameters of micellization, aggregate dimensions, or interfacial properties. Records were excluded if the IL was present only as a trace additive or impurity, if the study concerned surfactant behavior in water without an IL, if the IL served exclusively as a reaction medium, extractant, or electrolyte without analysis of amphiphile self-assembly, or if the item was a conference abstract, a patent, or another non-peer-reviewed report without primary data.

Screening was performed on titles and abstracts and then on full text, and the data extracted from the retained studies were the ionic liquid composition, the surfactant identity and purity, the water content, the temperature, the aggregation threshold and the method used to determine it, the aggregate morphology and the technique used to resolve it, and any reported thermodynamic or structural parameters. These fields form the columns of Table 4, so that the coverage of the literature is visible to the reader. Where several studies reported the same nominal system under different conditions, the conditions are preserved in Table 4 rather than pooled, because pooling them would produce a comparison that no single experiment supports.

Two limitations of the procedure should be stated. Selection was not blind and no formal risk-of-bias assessment was performed, so the synthesis depends on the judgment of the authors. In addition, the retained literature is subject to publication bias: studies reporting no aggregation, no measurable effect, or a negative result are rare in this field, which means that the frequency with which a particular mechanism is reported is not an unbiased estimate of its importance. This constraint is carried through to Section 5.7, where the reporting practices that would remedy it are discussed.

## 2. Structural Fundamentals of Surfactants and Ionic Liquids

### 2.1. Molecular Structure of Surfactants and Theoretical Basis of Aggregation

Surfactants are a typical class of amphiphilic molecules, whose structure consists of a hydrophilic headgroup and a hydrophobic tail chain [61]. This amphiphilic nature enables them to exhibit distinct interfacial activity in solution [62] and to spontaneously form ordered aggregates under certain concentration conditions, such as micelles, vesicles, microemulsions and lyotropic liquid crystals [63]. The occurrence of self-assembly is essentially driven by the minimization of the system’s Gibbs free energy, involving a comprehensive balance of various factors such as hydrophobic chain-to-chain interactions [64], electrostatic repulsion between headgroups [65], and solvation effects [66].

The critical micelle concentration (CMC) is a key parameter describing the aggregation behavior of surfactants, representing the concentration threshold at which monomers begin to transform into aggregates in large numbers [67]. Below the CMC, molecules exist primarily in monomeric form; once the CMC is exceeded, the concentration of free monomers in the system tends to stabilize, and newly added molecules preferentially participate in aggregate formation [68]. The morphology of the aggregates can be predicted using the critical packing parameter (*P*) [69]:

*P* = *v*/*al* (1)

where *v* is the volume of the hydrophobic chain, *a* is the effective cross-sectional area of the headgroup, and *l* is the fully extended length of the hydrophobic chain. Different ranges of *P* are associated with different aggregate geometries: *P* ≤ 1/3 corresponds to spherical micelles [70]; 1/3 < *P* ≤ 1/2 to rod-shaped or ellipsoidal micelles [71]; 1/2 < *P* ≤ 1 to lamellar or bilayer structures [72]; and *P* > 1 to inverse structures [73]. The packing parameter is, however, a mean-field geometric descriptor rather than a predictive theory, and its transfer to IL media requires care. Three limitations are particularly important.

*P* is defined for a dilute, weakly correlated solvent, and the effective headgroup area *a* is obtained indirectly, usually from surface-tension or conductivity data that assume a sharp aggregation threshold and ideal mixing at the interface [74]. In an IL, the aggregation threshold is often broad, and the interface is densely populated by solvent ions, so both assumptions are weak. *a* is not an intrinsic molecular constant. In IL media, it responds to counterion penetration, to specific ion pairing, and to the solvation structure of the headgroup, which means that a fitted value of *P* conflates molecular geometry with solvent effects and cannot be compared across systems unless the water content, temperature, and ionic composition are matched [75]. The model does not describe the intermediate regimes, curvature frustration, or the coupling between aggregation number and aggregate shape that arise when the interface is stiffened by hydrogen bonding or by π-stacking. Packing-parameter arguments are therefore used in this review as a qualitative organizing principle, and quantitative morphological claims are made only where they are supported by direct structural measurement (Section 4.1) or by molecular simulation with a validated force field (Section 4.2.4).

### 2.2. Structural Characteristics and Nanoscale Heterogeneity of Ionic Liquids

Ionic liquids (ILs) generally refer to organic salts with melting points below 100 °C [76], consisting of organic cations and inorganic or organic anions [77]. Unlike conventional molecular solvents, ionic liquids exhibit distinct structural heterogeneity at the nanoscale [78]. Experimental and simulation studies reveal that many ILs exhibit spatially segregated polar and non-polar domains with typical domain sizes of 1–3 nm, as evidenced by SAXS and MD simulations. This nanoscale partitioning arises from the hydrophobic aggregation of cationic alkyl chains [79], from Coulombic interactions between ion pairs [80], and from the reconfiguration of hydrogen-bond networks [81]. Two qualifications apply to these domain sizes. The values derived from scattering depend on the model used to fit the data, so they are apparent rather than absolute dimensions, and the degree of segregation is itself sensitive to the alkyl chain length, the anion, the temperature, and the residual water content [82]. Consequently, IL systems are not homogeneous continuous phases, but rather soft-matter solvents possessing an intrinsic nanoscale organizational structure. This structural characteristic enables them to exert a templating effect on the aggregation behavior of amphiphilic molecules [83].

### 2.3. Structural Control and Designability of Ionic Liquids

A core advantage of ionic liquids lies in their structural tunability [84]. By altering the length of the cationic alkyl chain, introducing functionalized substituents, or changing the type of anion, the following properties can be systematically modulated: polarity [85], viscosity [86], hydrogen-bonding ability [87], hydrophobic/hydrophilic ratio [88], and electrical conductivity [89]. For example, long-chain imidazolium cations are more prone to forming distinct non-polar nanodomains [90], thereby enhancing solvophobic-driven effects; conversely, strongly hydrogen-bonding anions may alter the solvation behavior of surfactant headgroups. Jafari-Chashmi et al. [91] investigated the mixed self-assembly behavior of imidazolium ionic liquids (ILs) with sodium N-laurylsarcosinate (NLS). The results indicated that the introduction of highly hydrogen-bonding anions significantly altered the solvation behavior of the surfactant headgroups, enhancing electrostatic attraction and steric repulsion between molecular components by promoting headgroup desolvation. This restructuring of the solvation layer led the system to exhibit strong synergistic effects [92]; the experimentally determined interaction parameter (βm) exhibited a high negative value, and a tendency towards 1:1 molecular pairing was observed within the mixed micelles. This study demonstrates that ILs not only participate in the system as solvents but also directly influence the self-assembly pathways of surfactants through their ionic structure. This ‘solvent-structure coupling modulation’ is a key feature that distinguishes IL systems from traditional solvent systems [93].It also illustrates why structure–property correlations in this field must be established for defined cation–anion pairs rather than for ILs as a class.

### 2.4. Protic and Aprotic Ionic Liquids

The distinction between protic ionic liquids (PILs) and aprotic ionic liquids (AILs) is frequently noted but seldom followed through to its consequences for self-assembly, even though the two families differ in the properties that matter most for aggregation: the availability of a mobile proton, the density and lifetime of hydrogen bonds, the extent of ion pairing, and the degree of nanoscale segregation.

PILs are formed by proton transfer from a Brønsted acid to a Brønsted base and therefore contain a proton that is available for hydrogen bonding. The resulting networks are dense and dynamic, with hydrogen-bond lifetimes long enough to organize the solvent but short enough to permit rapid structural reorganization [94,95]. This dual character has two consequences for amphiphile self-assembly. The polar network offers strong, directional interactions to the surfactant headgroup, which tends to stabilize the dispersed monomer and therefore to raise the CMC unless the aggregate interface can also accept hydrogen bonds. At the same time, the alkyl chains of the IL cation segregate from the polar network and form well-defined non-polar domains that accommodate the surfactant tail at relatively low energetic cost [96]. Protic media such as ethylammonium nitrate are consequently characterized by a favorable solvophobic driving force coupled to strong competing solvation of the headgroup, and zwitterionic and nonionic amphiphiles are often better able to assemble in them than strongly charged surfactants whose headgroup charge demands direct charge compensation [97].

AILs have no exchangeable protons, and their hydrogen bonding is confined to interactions between the cation and the anion. Their cohesion is dominated by Coulombic attraction modified by alkyl-chain segregation, and their ionicity, that is, the fraction of ions that are unpaired and free to act as independent charge carriers, varies widely with cation and anion structure [11,56]. Because the driving force for aggregation in an AIL is largely solvophobic and electrostatic rather than hydrogen-bonded, the CMC responds more strongly to the length of the cation alkyl chain and to the ion-pairing strength of the anion, while the aggregate morphology is more sensitive to charge screening at the headgroup [42,49,57].

The practical implication is that mechanistic statements cannot be transferred between the two families. A hydrogen-bond-mediated stabilization pathway established in a PIL does not apply to an imidazolium-based AIL, where the same interaction is absent, and a screening-dominated pathway characterized in an AIL cannot be assumed in a PIL, where headgroup hydrogen bonding competes with charge compensation. Table 6 summarizes the differences and the assembly consequences that follow from them, and the distinction is maintained in the mechanistic discussion in Section 3.

### 2.5. Trace Water in Nominally Anhydrous Ionic Liquids

Water is never entirely absent from an IL system unless the IL has been rigorously dried, and the concentrations that remain after routine handling are large enough to change self-assembly behavior. This section therefore treats water as a variable rather than as an impurity to be ignored, and it makes explicit the water content of the systems discussed in the remainder of the review.

The residual water content of an as-received IL depends strongly on the cation–anion pair and on handling. Hydrophilic ILs such as imidazolium halides and nitrate salts are hygroscopic and readily equilibrate with atmospheric moisture, whereas ILs with fluorinated anions such as bis(trifluoromethylsulfonyl)imide are appreciably more hydrophobic. Reported values for nominally dry samples span several orders of magnitude, from below 10 ppm to thousands of ppm, depending on the IL and on the drying protocol [98]. Reporting the water content is therefore not a formality: without it, the comparison of CMCs between studies is not well defined.

Water alters IL structure on several length scales. It is incorporated into the polar network, where it competes for hydrogen-bond donor and acceptor sites and can disrupt the charge alternation that organizes the ionic matrix [99,100]. Added water can nevertheless enhance the segregation between polar and non-polar domains, and the water molecules themselves cluster into small polar pockets rather than dispersing homogeneously. The viscosity of the IL decreases sharply with increasing water content, which changes both the rate at which aggregation equilibrium is reached and the interpretation of dynamic measurements such as diffusion and relaxation experiments [86]. Water also raises the effective dielectric permittivity of the medium and provides mobile protons, so that electrostatic screening and hydrogen bonding are both modified [73,99].

For self-assembly, these changes translate into three effects that are frequently observed but rarely separated. Small additions of water usually lower the CMC, because the amphiphile is transferred from a less hospitable medium into a more water-like one and because the solvophobic driving force increases as the polar network is disrupted. The aggregate morphology can change as well, with lyotropic phases appearing or disappearing as the water content crosses a threshold; the water-driven phases reported for long-chain imidazolium iodides probed by neutron scattering illustrate this behavior [60]. The dynamics of the system change through the coupling of the aggregate to the solvent viscosity [86,101]. The magnitude of these effects depends on the water-to-surfactant ratio rather than on the nominal purity of the IL alone, so a system described as anhydrous can behave as a mixed aqueous–IL system once the surfactant concentration falls below the water concentration.

Water content should therefore be reported as an absolute concentration determined by Karl Fischer titration, measured on the same batch that is used in the self-assembly experiment, and stated alongside the surfactant concentration so that the water-to-surfactant molar ratio can be calculated. Table 4 includes a column for this quantity, and studies that do not report it are identified there, because the corresponding comparisons remain provisional until the value is supplied.

## 3. Mechanisms of Surfactant Self-Assembly in Ionic Liquids

### 3.1. Reconfiguration of Self-Assembly Driving Forces in Ionic Liquid Environments

Before the individual driving forces are examined, the status of the mechanistic framework used in this review should be stated. The framework is a schematic organizing device, and the diagrams that illustrate it are drawn to show how the contributing interactions are arranged relative to one another rather than to reproduce any measured system. Where a mechanistic statement is supported by direct experimental measurement or by validated simulation, this is said explicitly and the supporting reference is given; where it is an interpretation of schematic evidence or a generalization across systems of different chemical composition, it is identified as a hypothesis. This distinction matters because the relative weight of the driving forces varies with the IL family, the surfactant class, the water content, and the temperature, so a mechanism established in one system is not automatically transferable to another.

In conventional aqueous systems, surfactant self-assembly is primarily driven by the hydrophobic effect, whereby the aggregation of non-polar chains minimizes disruption to the hydrogen-bond network of water molecules [52]. In ionic liquid (IL) environments, this classical framework becomes insufficient because the continuous phase is no longer a structurally homogeneous molecular solvent [55]. The aggregation behavior of surfactants in ILs is therefore more appropriately understood as the result of synergistic modulation by multiple interactions driven by solvophobic forces [102]. These four factors do not act independently, and it is useful to distinguish the role that each plays at different stages of assembly. In the simplest case, solvophobic interactions establish whether aggregation is favourable at all, electrostatic screening modulates how closely the headgroups can approach once aggregation has begun, hydrogen bonding and specific ion interactions determine which of the available interfaces is the more stable, and van der Waals interactions between chains select the packing geometry. This sequence is a heuristic rather than a universal mechanism, and it fails in specific and identifiable ways.

The most important qualifications are the following. In strongly hydrogen-bonded protic ILs, the hydrogen-bond network is not a downstream stabilizing factor but a primary determinant of whether aggregation occurs, and the solvophobic and hydrogen-bond contributions cannot be separated experimentally (Section 2.4). In ILs of low ionicity, charge screening is weak and the electrostatic contribution is dominated by specific ion pairing rather than by collective screening, so the second stage of the sequence is absent. For nonionic and zwitterionic amphiphiles, the electrostatic term largely disappears, and the morphology is controlled instead by the balance between solvation of the headgroup and chain packing. For surfactants with aromatic headgroups or with chains capable of π-stacking, the ordering of the last two stages is reversed, because van der Waals and π-stacking interactions modify the interface before the hydrogen-bond environment is reorganized. Finally, in IL–water mixtures the hydrophobic effect re-emerges as a leading term and the whole sequence is replaced by the aqueous one perturbed by ion-specific effects (Section 1.2). The framework is therefore best read as a checklist of contributions whose relative magnitudes must be established for each system, rather than as a reaction pathway. The following subsections examine each contribution and indicate where the evidence is direct and where it is inferential.

**Figure 1:**
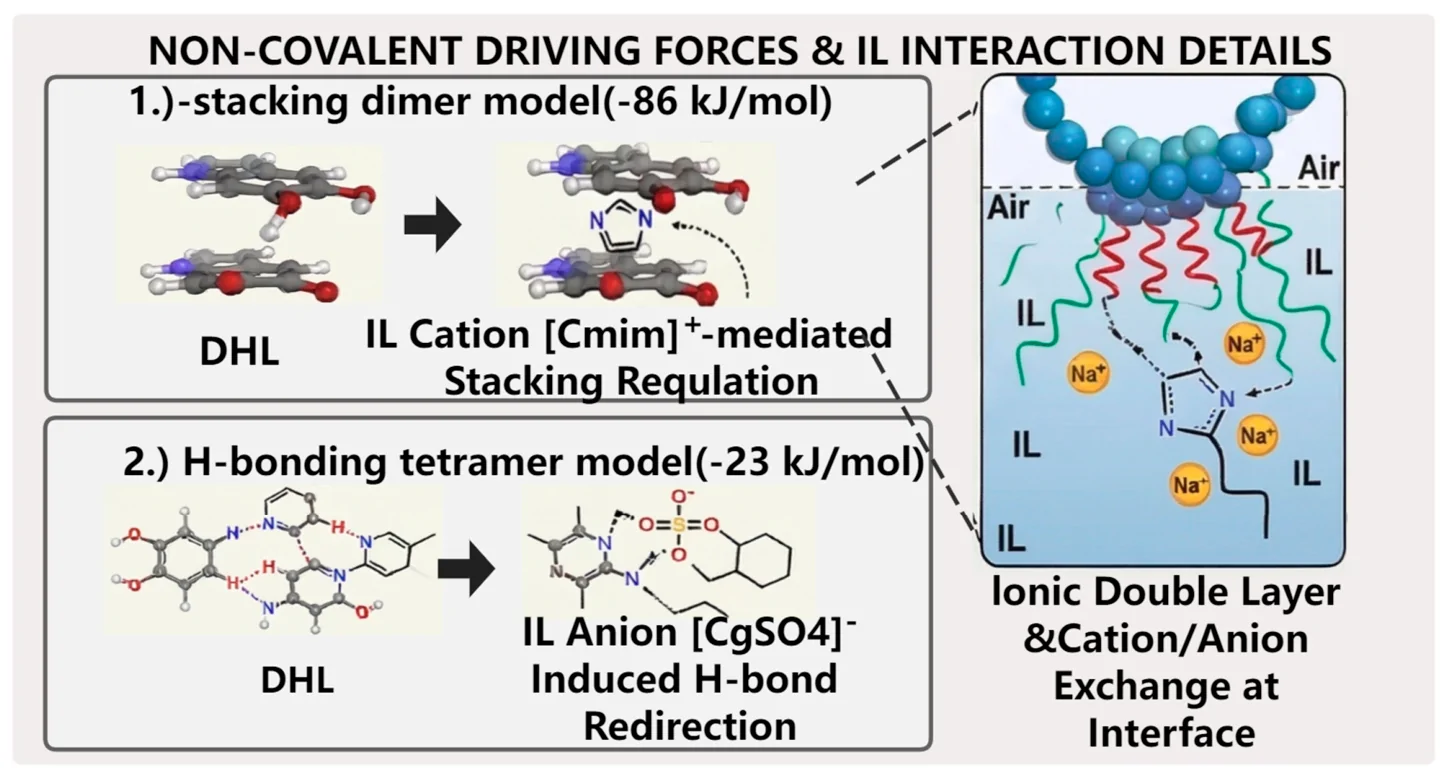
Self-assembly mechanisms of surfactants in ionic liquids: from molecular building blocks, non-covalent driving forces and interfacial modulation to morphological evolution and rheological behavior. Reproduced with permission from Ref. [55], copyright 2023, Royal Society of Chemistry.

(1) The solvophobic effect and local solvation effects determine the compatibility between hydrophobic segments and the microstructure of the ionic liquid, thereby influencing whether aggregation occurs and at what critical concentration [103]. This process is illustrated through the selection of different building blocks, such as dodecyltrimethylammonium bromide (DTAB), which exhibits minimal steric hindrance, and didodecyldimethylammonium bromide (DDAB) and dodecylmethylpyrrolidinium bromide (DMPB), which possess π-stacking capability, so as to adapt to the complex microenvironment of the ionic liquid solvent (see Section 3.2 for details).

(2) The high-ionic-strength background and counterion penetration weaken the Coulombic repulsion between the headgroups of ionic surfactants, reducing the effective headgroup area and promoting closer molecular packing [104]. As illustrated by the interfacial modulation process, under the synergistic shielding effect of NaCl or ionic liquid ions, the double layer collapses, and the effective headgroup area a0 decreases, accompanied by a rapid decrease in the interfacial tension observed experimentally (see Section 3.3 for details).

(3) The hydrogen-bond network prevalent within ionic liquids can modulate the thermodynamic equilibrium of self-assembly through two mechanisms: stabilizing aggregate interfaces or preferentially solvating monomers [105]. According to the non-covalent interaction model shown in Figure 1, the ionic liquid anion [C_8_SO_4_]^−^ induces hydrogen-bond reorientation and assembles a tetrameric structure with an interaction energy of approximately −23 kJ mol^−1^, thereby contributing to the stabilization of aggregates at the thermodynamic level (see Section 3.4 for details).

(4) Van der Waals interactions between hydrophobic chains, π-π stacking, and the resulting effect on the critical packing parameter further determine the curvature, size, and phase evolution of the aggregates. As shown in Figure 1 π-stacking dimers mediated by the ionic liquid cation provide a strong driving force of −86 kJ mol^−1^, causing the critical packing parameter to evolve from below 1/3 to approach 1/2 or even 1 and driving the morphological transition from spherical micelles and vesicles to worm-like micelles. In the DMPB system in particular, the headgroup stiffening induced by π-π stacking ultimately leads to the entanglement of worm-like micelles and to their characteristic rheological response (see Section 3.5 for details).

It is thus evident that self-assembly in ILs is not a process triggered by a single driving force, but rather the result of a multi-level coupling involving aggregation initiation, aggregation enhancement, structural stabilization, and morphological selection [106]. It is worth emphasizing that, although the schematic diagrams summarize unified assembly rules, the strength of each interaction varies across systems. Solvophobic effects are intensified in long-chain cationic ILs, whether protic or aprotic; electrostatic shielding operates only for ionic and zwitterionic surfactants; hydrogen-bond networks dominate protic ionic liquid systems yet remain weak in aprotic imidazolium ILs; and van der Waals stacking exerts stronger modulation on long-chain and aromatic surfactants in all IL media. The finding that different types of ionic liquid induce significantly different critical micelle concentrations, aggregation numbers, and structural types is not merely a consequence of differences in polarity, but is more closely related to their ionic structure [69], hydrogen-bonding ability [107], nanoheterogeneity [108] and viscosity [109]. Ionic liquids can therefore be regarded not merely as alternative solvents distinct from water, but as structural media capable of actively modulating self-assembly pathways through ion design. The following sections discuss these four aspects in detail.

### 3.2. Solvophobic Effect and Solvation Effects

Solvophobic effect refers to the unfavorable contact between surfactant tails and polar IL networks, driving aggregation to minimize this contact. Solvation effect, in contrast, describes the stabilizing interaction between IL ions and surfactant headgroups/monomers, which can either promote or hinder aggregation depending on the IL structure [38]. Unlike the classical scenario in aqueous solutions, where non-polar segments are repelled by water, ionic liquids often exhibit a dual nature, containing both polar domains formed by ionic headgroups and non-polar domains formed by alkyl chains [56]. Upon entry into the system, the hydrophobic chains of surfactant monomers may either be partially accommodated by local non-polar domains or repelled upon contact with the polar network. Consequently, the aggregation process in ILs is more appropriately clarified as a molecular rearrangement driven by a solvophobic effect: when compatibility between the hydrophobic chains and the microstructure of the ionic liquid is poor, the system tends to minimize the area of unfavorable contact through aggregation, thereby lowering the total Gibbs energy.

The structural pathways of surfactant self-assembly are profoundly regulated by the localized nanostructure and cohesive network of ionic liquid media. In protic ionic liquids (PILs), the dominant driving forces for aggregate formation originate from dense, dynamic hydrogen-bonded matrices, which induce prominent solvophobic effects and separate polar networks from non-polar alkyl segments [94]. As schematically demonstrated by the microscale network, this structural model visualizes the spatial distribution of amphiphilic moieties. Cohesive hydrogen bonds and intense electrostatic interactions dominate the arrangement of polar headgroups, whereas non-polar hydrophobic tails are selectively solvated within IL nanodomains through van der Waals forces and π-π stacking interactions.

Further theoretical analyzes clarify that the geometric configuration and energy distribution of these aggregates are collectively determined by the subtle interplay of interionic hydrogen bonding and dispersion forces [99]. Such synergistic intermolecular interactions dynamically adjust interionic distances in response to variations in the surrounding chemical microenvironment. It is thus evident that, within the IL, the microenvironment occupied by the surfactant monomers is not a static background but rather undergoes dynamic reorganization in response to changes in the ionic liquid structure.

**Figure 2:**
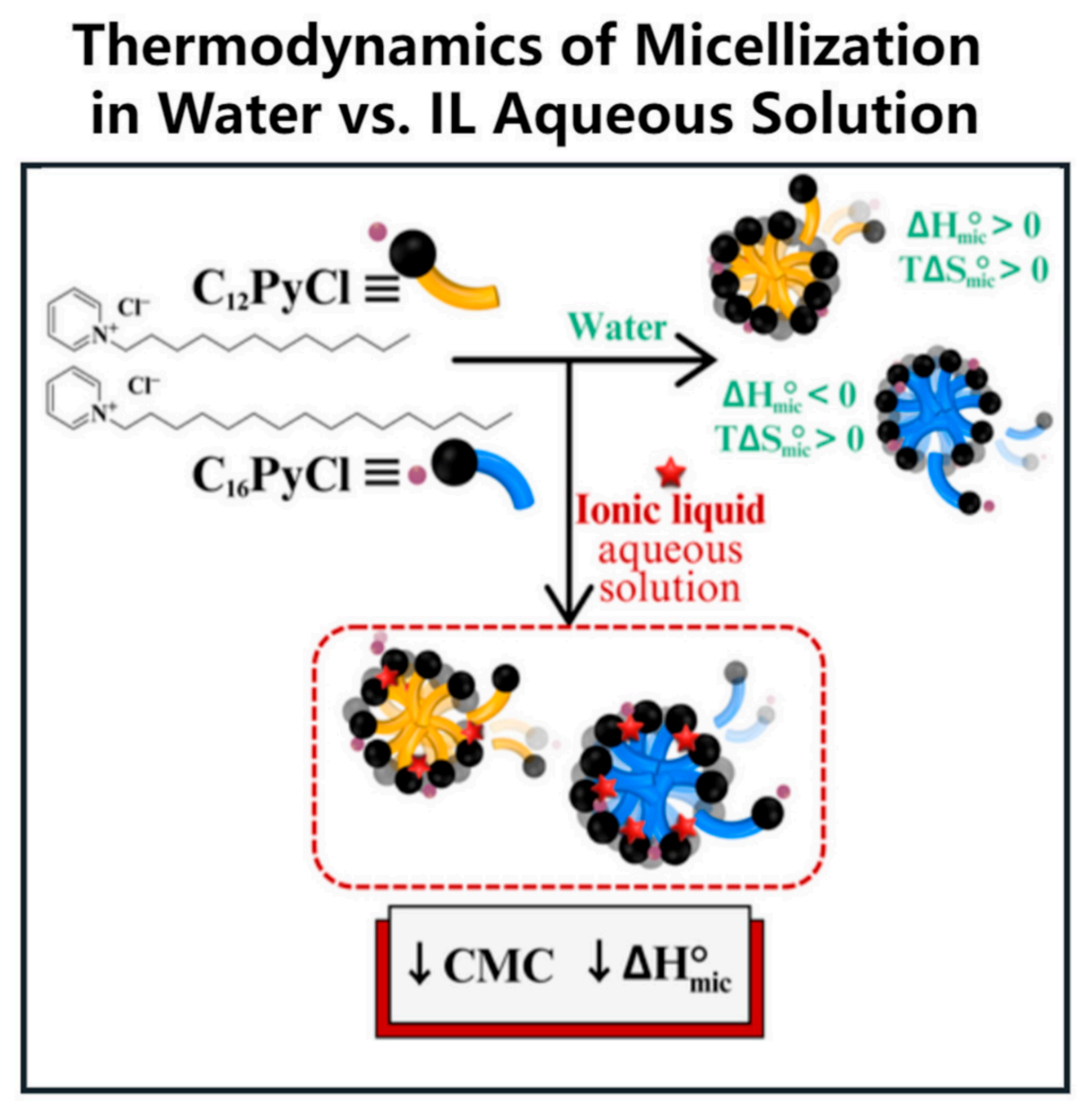
Modulation of surfactant micellization by ionic liquid solvophobicity and solvation effects: from molecular interactions to morphological control. Reproduced with permission from Ref. [57], copyright 2024, John Wiley and Sons.

These dynamic solvation variances exert a direct control on the critical micelle concentration (CMC) and the overall thermodynamics of the nucleation process. For instance, in aprotic imidazolium-based systems, an increase in the tailored hydrophobicity of either the structural ionic liquid components or the packed amphiphilic chains significantly lowers the thermodynamic barrier for assembly [57]. As schematically mapped out in the thermodynamic profiles of Figure 2, this hydrophobicity-driven behavior is clearly demonstrated by isothermal titration calorimetry (ITC) and conductivity metrics, which confirm that enhanced system hydrophobicity systematically reduces the CMC threshold while simultaneously amplifying the exothermic response (ΔH°_mic_ < 0). This key thermodynamic shift reveals that the structured ionic liquid matrix acts as an active driving medium, optimizing the localized coordination around the non-polar surfactant tails through enhanced dispersion forces and enthalpy-favorable preferential solvation layers. In contrast, the self-assembly patterns of nonionic amphiphiles rely heavily on a competitive balance between bulk structural stabilization and monomer solvation [47]. Variations in the specific ionic liquid anions can drastically shift both the initial aggregation thresholds and the macroscale dimensions of the aggregates. Furthermore, as illustrated in the proposed molecular assembly models systematically expanding the alkyl chain length on the ionic liquid cation introduces distinct steric mismatches and solvent-tuned regimes. In low-concentration or short-chain IL matrices, the system maintains a looser water-like or simplified alignment; however, transitioning to high-concentration or long-chain IL scenarios triggers deep z-chain infiltration and enhanced network shielding that directly reconfigures critical phase properties, such as the cloud point and individual micellar size-stability [110]. This dual response strongly underlines that ionic liquids do not operate as inert background environments, but rather serve as active structural design components that reconfigure the self-assembly pathways through localized non-covalent interactions. Overall, when the ionic liquid has a weaker ability to solvate hydrophobic chains, or when its nanostructure causes hydrophobic chains to be more inclined to escape the polar environment, surfactants typically aggregate more readily and exhibit a lower CMC; conversely, if the IL can effectively stabilize the local environment around the monomers, the driving force for aggregation is correspondingly weakened. It should be noted that this process is not governed by a single physical property parameter but is the result of the combined effects of the ionic structure, nanoheterogeneity and local solvation capacity of the ionic liquid.

### 3.3. Electrostatic Screening and Counterion Penetration in IL Media

For ionic surfactants, electrostatic repulsion between headgroups is typically one of the key factors limiting aggregate formation and further growth [111]. In aqueous solutions, this repulsion can usually be partially mitigated by the addition of inorganic salts or counterions [112]. Furthermore, in aqueous mixed surfactant systems, headgroups possessing opposite charges can exhibit strong Coulombic attractions, leading to highly aggregated and well-ordered arrangements at the interface [54]. This behavior highlights that charge neutralization and the subsequent reduction in the effective headgroup area serve as critical factors in lowering the thermodynamic barrier for self-assembly nucleation. However, in ionic liquid systems, as the continuous phase itself is a high-concentration ionic medium, the electrostatic environment differs fundamentally from that of conventional dilute solutions [53]. The abundance of anions and cations not only increases the overall ionic strength of the system but also allows them to penetrate the vicinity of the surfactant headgroup layer. Through mechanisms such as counterion penetration, local charge compensation and a reduction in effective charge density, they significantly weaken the Coulombic repulsion between headgroups [113]. The direct consequence is a reduction in the effective headgroup area, an increase in molecular packing density, and a further alteration of the system’s critical packing parameters.

**Figure 3:**
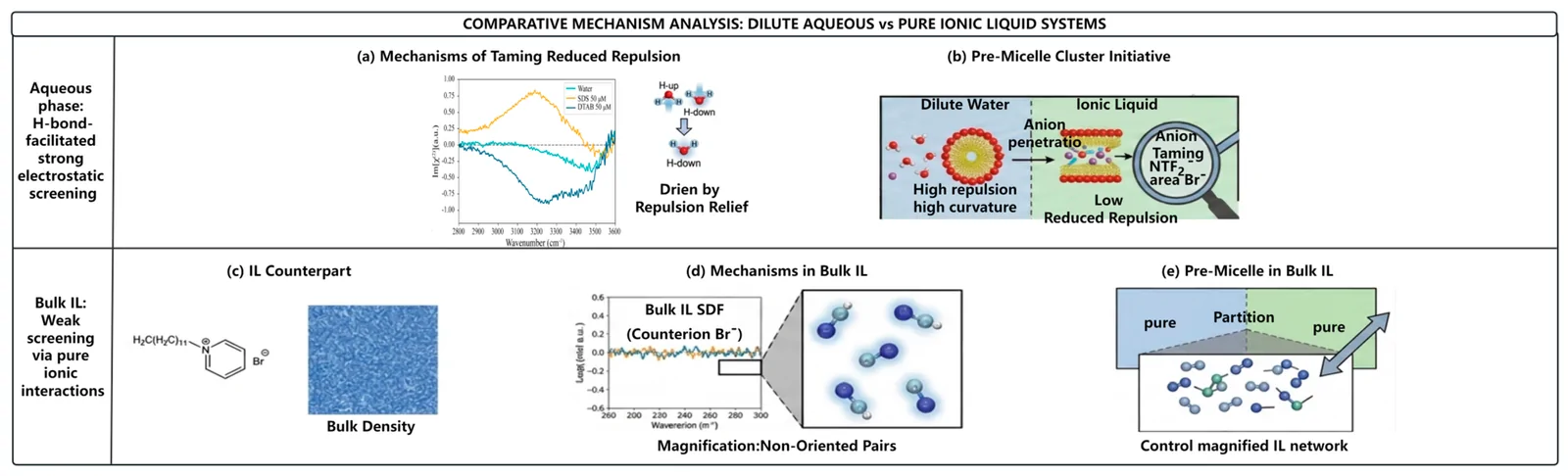
Comparative electrostatic screening and surfactant assembly in water and in ionic liquids. (**a**) Reproduced with permission from Ref. [54], copyright 2024, American Chemical Society. (**b**) Reproduced with permission from Ref. [49], copyright 2024, Royal Society of Chemistry.

As systematically compared in the schematic of Figure 3, the electrostatically driven adsorption and subsequent phase sequences manifest fundamentally different mechanisms when transitioning from a dilute aqueous medium to a bulk ionic liquid environment. In conventional dilute aqueous solutions, electrostatic repulsion between charged surfactant headgroups is the primary thermodynamic barrier to aggregation. Upon addition of inorganic salt or of counterions that bind specifically, that repulsion is attenuated. At low added-salt concentrations, the effect is described adequately by the Debye-Hückel treatment of the electrical double layer, in which a diffuse cloud of hydrated counterions screens the micellar surface over a length that decreases as the ionic strength increases, reducing the effective headgroup area a_0_ and permitting micellar growth.

The Debye–Hückel description is a limiting law for dilute electrolytes, and it cannot be transferred to a bulk IL. In a typical IL the ion concentration is of the order of 5–8 mol L^−1^ and the mean ion spacing is comparable to or smaller than the ion diameter, so the calculated screening length falls below the physical size of the ions and the assumptions of the model, namely point charges in a continuum dielectric, weak coupling, and negligible ion-ion correlation, all fail [11]. Screening in these media is better described in terms of explicit ion correlation and ion pairing, and the relevant quantities are the ionicity, that is, the fraction of ions that are dissociated rather than bound in pairs or clusters, the Bjerrum length, and the strength of the specific cation–anion interaction, which depends on charge distribution, polarizability, and hydrogen bonding [11,15,56]. Mean-spherical-approximation and restricted-primitive-model treatments capture part of this behavior, and molecular simulation is generally required when the interface is spatially structured [11,82]. The term screening is therefore used in this review in a phenomenological sense, meaning the reduction in the net electrostatic repulsion experienced by the headgroups, which in an IL arises from local charge compensation by penetrating ions rather than from a diffuse double layer. Physical parameters derived from the Debye–Hückel model, such as a Debye length or a screening constant, are not applied to neat IL systems, and the underlying mechanism is described instead in terms of ion penetration, ion pairing, and charge correlation.

In sharp contrast, as depicted in the bulk ionic liquid counterpart, a pure IL medium represents a high-concentration, solvent-free ionic matrix where traditional long-range double-layer treatments break down. Here, rather than acting as distant screening agents in water, the constituent anions and cations of the bulk IL directly penetrate the immediate vicinity of the surfactant headgroup layer. For instance, Pan et al. [49] demonstrated that in aprotic ionic liquids (AILs), the spontaneous penetration of IL anions near imidazolium surfactant heads induces local charge compensation, effectively ‘taming’ the repulsion. This localized charge-directed orientation under weak, ion-only screening triggers a much tighter molecular packing density. Consequently, while ionic surfactants in dilute water typically trace a high-curvature path toward spherical micelles, the severe contraction of a_0_ in pure IL environments drives a direct, single-phase sequence evolution from pre-micellar clusters straight toward low-curvature, macroscopic continuous lamellar phases or highly ordered liquid crystal structures, even without the assistance of molecular water [42].

### 3.4. Hydrogen-Bonding Networks and Molecule-Specific Interactions

**Figure 4:**
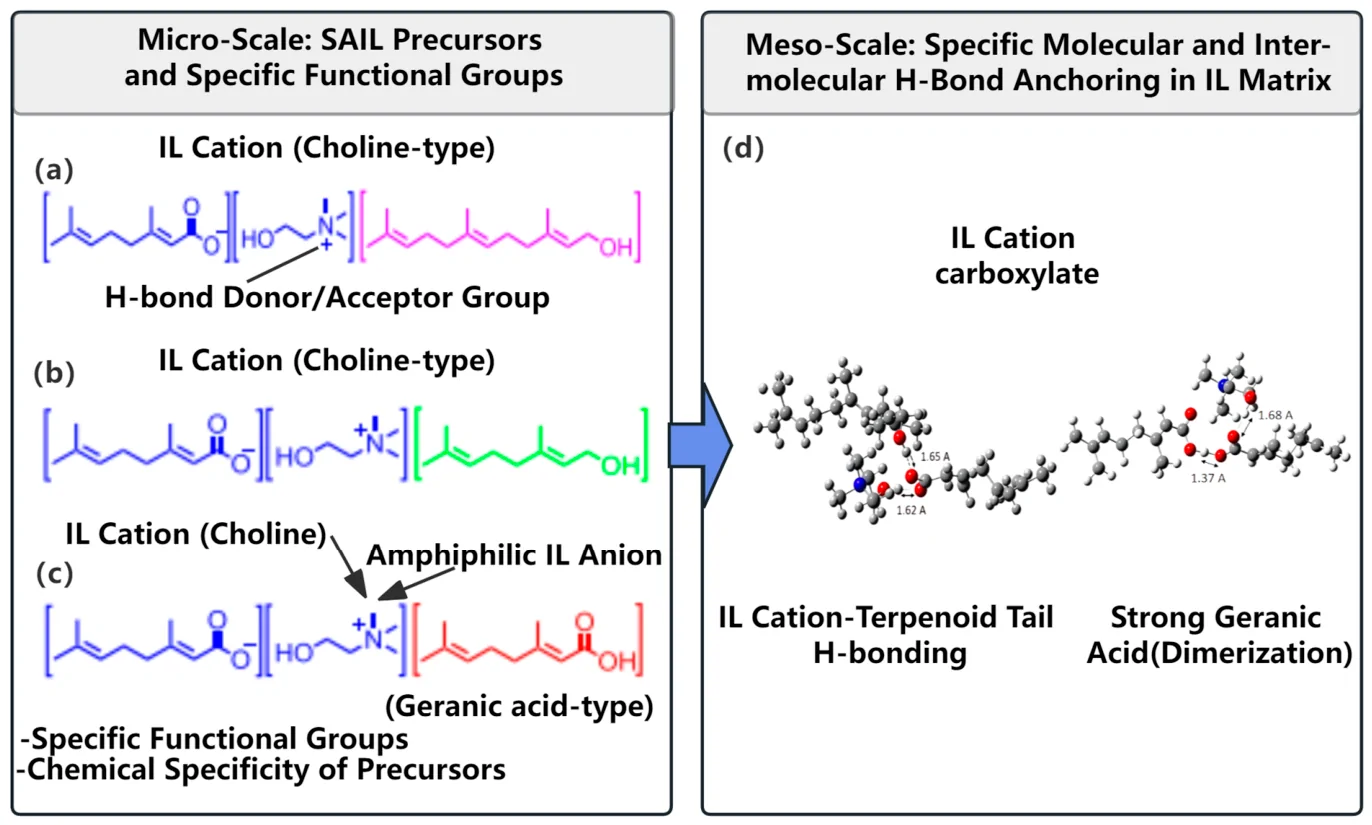
Ionic liquid-mediated surfactant self-assembly: molecular interactions and hydrogen-bonding mechanisms. (**a**–**d**) Reproduced with permission from Ref. [100], copyright 2025, American Chemical Society.

In addition to electrostatic interactions, hydrogen-bonding networks represent another key feature that distinguishes ionic liquids from conventional molecular solvents. Many ionic liquids, particularly protic systems and those containing structural units such as imidazolium and choline, often possess a wealth of hydrogen-bond donor and acceptor sites within their structures, thereby forming dynamic yet organized hydrogen-bonding networks [58]. As illustrated in Figure 4a–c, precursors containing specific hydrogen bond donors and acceptors are shown; the electrostatic potential surface plots visually represent these specific acceptor and donor sites as red and blue regions, respectively, forming the structural basis of complex hydrogen bonding networks. This network not only influences the microstructure and rheological properties of the ionic liquid itself but also significantly alters the local interactions at the interfaces between surfactant monomers and aggregates. Therefore, when discussing self-assembly mechanisms in IL systems, hydrogen bonding should not be regarded merely as a secondary factor but understood as a decisive variable determining local stability and molecular recognition selectivity [59].

The influence of hydrogen bonding on self-assembly exhibits a distinct duality. On the one hand, if the ionic liquid components can form stable hydrogen bonds with surfactant headgroups, interfacial regions, or aggregate shells, this may reduce the interfacial energy of the aggregates, enhance local structural order, and improve the thermodynamic stability of the aggregates. Research by Marlow et al. [96] on ethylammonium nitrate (EAN) systems demonstrated that the distribution of hydrogen bond donors and acceptors within the ionic liquid influences the effective surface area and local solvophilicity of surfactant headgroups during self-assembly, indicating that hydrogen bond networks can participate in aggregate modulation by modulating the structure of the interfacial layer (Figure 4d). This anchoring tightly bridges the IL matrix with the surfactant molecules, greatly enhancing the local order of the aggregates. Simulation results by Pal et al. [114] on carboxyl-functionalized imidazolium ionic liquids further indicate that Coulombic and hydrogen-bond interactions jointly maintain a relatively stable bulk network, providing a stable molecular-level foundation for the formation and maintenance of aggregate structures.

On the other hand, hydrogen bonding may also hinder aggregation. If the ionic liquid preferentially forms a stable solvation shell with the surfactant monomer, thereby conferring a high degree of local stability to the monomer in the dispersed state, the free energy gain from aggregation will correspondingly decrease, resulting in an increase in the critical micelle concentration (CMC) or a reduction in the aggregation number. Research by Tamate et al. [115] on ionic liquid-modulated ionic gels of block copolymer micelles indicates that competitive hydrogen bonding between anions, cations and polymer chains weakens the intermicellar network connections and alters the viscoelasticity of the system, suggesting that excessively strong ionic liquid–monomer interactions may be detrimental to the formation of higher-order self-assembled structures. Research by Oluwole et al. [100] on ionic liquid mixed solvents further points out that increased hydrophobicity in the system does not necessarily weaken hydrogen bonding; on the contrary, it may enhance the hydrogen bonding network through local environmental reorganization. This suggests that hydrogen bonding and solvophobicity do not simply compete with one another but may instead exhibit synergistic coupling characteristics.

Consequently, the pivotal role of the hydrogen-bond network does not lie in simply promoting or inhibiting self-assembly, but rather in determining which stability pathway the system favors: whether it prioritizes the stability of dispersed monomers or that of aggregate interfaces. When the former predominates, aggregation is delayed; when the latter predominates, self-assembly occurs more readily and exhibits greater stability. It is precisely for this reason that different anions, cations with varying hydrogen-bonding capabilities, and the introduction of water or other cosolvents can all significantly alter the final aggregation behavior [48]. This molecular specificity inherent in the hydrogen-bond network is also a key reason why IL systems cannot be fully characterized by a single empirical rule [95].

### 3.5. Modulation of Van Der Waals Interactions and Packing Parameters in Hydrophobic Chains

**Figure 5:**
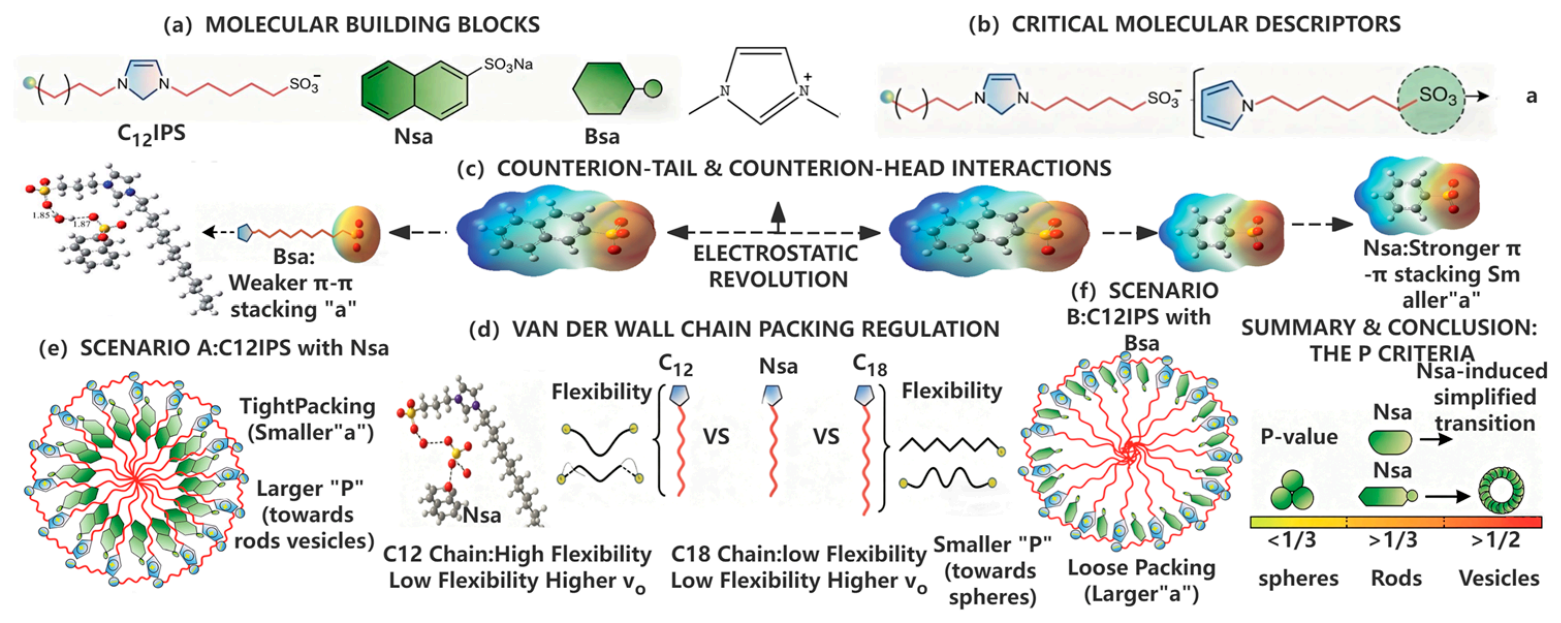
Modulation of surfactant packing and morphology in ionic liquids: the role of π-π interactions and van der Waals forces. (**a**–**f**) Reproduced with permission from Ref. [43], copyright 2016, Royal Society of Chemistry.

Although the ionic liquid environment introduces a variety of new modulating factors, van der Waals (vdW) interactions between hydrophobic chains remain an indispensable fundamental driving force for the formation of aggregate cores in surfactants [116]. Particularly in systems with longer chains, a higher number of chains, or aromatic structural units, intermolecular dispersion forces and π-π stacking can further enhance the attraction between chain segments, making the system more prone to forming dense aggregates (Figure 5a,c). Compared with the solvophobic effect and electrostatic shielding discussed above, this effect primarily governs the post-aggregation packing behavior and the final morphology. The chain-length effect provides the most intuitive illustration: as the hydrophobic chain length increases, interchain dispersion interactions strengthen and nucleation of aggregates is facilitated. This is typically manifested by a decrease in the critical micelle concentration (CMC), an increase in the aggregation number (Nagg), and an increase in micelle size. Yao et al. showed for glycosylated zwitterionic surfactants that extending the hydrophobic chain significantly enhances van der Waals attractions, leading to a simultaneous increase in both the aggregation number and the particle size [51]. Similarly, the study by Zhao et al. on double-chain imidazolium ionic liquid surfactants showed that, compared with single-chain systems, the double-chain structure can achieve self-assembly at lower concentrations owing to its greater chain-stacking capacity [117]. The geometric configuration of the hydrophobic end is therefore a key factor determining aggregation efficiency and stability. Sun et al. further indicated for zwitterionic surface-active ionic liquids (SAILs) that the enhanced van der Waals interactions caused by long chains, combined with π-π stacking between imidazole rings, collectively increased the molecular packing density and thereby significantly lowered the CMC [43].

In IL systems, however, the outcome of chain packing depends not only on the chain length and the number of chains but is also modulated by the ionic liquid environment through its effect on the effective headgroup area, the hydrophobic chain length, and the interfacial curvature (Figure 5d). It should be noted that the CPP framework is used here as a conceptual tool rather than a deterministic rule. In IL media, the same surfactant can adopt different morphologies depending on the IL cation/anion structure, water content, concentration, and temperature. Therefore, CPP-based predictions should be validated by scattering, spectroscopic, and simulation results. The critical packing parameter in ILs is often no longer merely an intrinsic parameter of the surfactant molecular structure, but rather the comprehensive result of molecule–solvent interactions. When electrostatic shielding reduces a, or when local solvation and hydrogen-bond networks make chain segments more prone to extension, the value of P may increase, and the system is more likely to transition from spherical micelles to rod-like micelles, lamellar phases, or even inverse phase structures (Figure 5e,f). Kawai et al. showed for the stacking behavior of quaternary ammonium-type ionic liquid lamellar phases that different headgroup topologies and alkyl chain arrangements significantly alter the effective headgroup area and the chain extension length, thereby inducing the formation of ordered lamellar structures with smaller interlamellar distances and denser packing [118]. In IL systems, the packing parameter is therefore essentially the result of environment-sensitive modulation rather than a simple geometric constant. Hydrophobic chain van der Waals interactions and the modulation of packing parameters jointly determine the ultimate self-assembly morphology in IL systems. When interchain attraction is weak and the headgroup area is large, the system tends to form small micelles with high curvature; when chain packing is enhanced and the effective headgroup area is reduced by electrostatic shielding or specific interactions, the system is more likely to transition towards low-curvature rod-like, lamellar, or liquid-crystalline structures. It is precisely for this reason that non-spherical micelles, worm-like micelles and lyotropic liquid crystals are often more readily observed in ionic liquids, providing a theoretical basis for achieving controllable morphologies through molecular and ionic design [119].

In summary, the self-assembly of surfactants in ionic liquids should not be viewed simply as a direct extension of the mechanisms observed in aqueous systems, but rather as a synergistic process shaped by multiple interactions: (1) Solvophobic effect and local solvation effects determine whether aggregation possesses sufficient thermodynamic driving force, serving as the starting point for self-assembly; (2) high ionic strength and electrostatic shielding weaken the repulsion between ionic headgroups, acting as key factors that promote aggregate growth and densification; (3) hydrogen-bond networks and the resulting molecule-specific interactions determine whether the system tends towards dispersion or ordered aggregation by modulating the relative stability of monomers and aggregates; while (4) van der Waals (vdW) forces between hydrophobic chains, π-π stacking, and changes in critical packing parameters modulated by the environment ultimately control the size, curvature and phase evolution of the aggregates.

Compared with traditional aqueous systems, the most distinctive feature of IL systems lies in the fact that their self-assembly driving forces are more diverse; the solvent is no longer merely a passive background but directly participates in structure formation; aggregate structures are highly sensitive to the specific anion–cation composition; and interactions between different forces are often not simply additive but exhibit significant synergy and competition. Therefore, ionic liquids provide a platform for the precise modulation of surfactant self-assembly through ionic design. Should it be possible in the future to establish quantitative correlations between ionic structure–local interactions–packing parameters–aggregate morphology, this is expected to propel the field from empirical observation towards a stage of predictable, design-driven structural construction. This mechanistic pathway will be revisited in Section 4.1.3, where specific LLC phase geometries are described.

### 3.6. Correlation Between Assembly Driving Forces and Aggregate Morphological Evolution

In summary, the morphological manifestation of surfactant aggregates within ionic liquid matrices is dictated by the thermodynamic requirement of system free energy minimization, where the spatial dimensions of the building blocks are cooperatively modulated by solvophobic effects, electrostatic screening, hydrogen-bond networks, and van der Waals forces. The energetic interplay among these localized non-covalent forces acts as a deterministic mechanism that dynamically regulates the effective headgroup area and hydrophobic tail volume, causing the critical packing parameter to deviate significantly from standard aqueous models. Consequently, any structural variation in the continuous ionic matrix alters the local energetic landscape and is immediately translated into a physical deformation of the aggregate interface.

This solvent-structure coupling serves as the explicit link bridging microscopic thermodynamic drivers with the macroscopic aggregate properties discussed next. Because each phase morphology represents a specific geometric manifestation of these coupled interactions, material performance cannot be predicted without a fundamental resolution of the physical dimensions involved. To establish this structure–property relationship, Section 4 extends the theoretical framework established here. It systematically categorizes the diverse macroscopic aggregate morphologies observed experimentally, while simultaneously addressing the experimental and simulation challenges associated with resolving these highly viscous, nanostructured non-aqueous fluids.

### 3.7. Quantitative Description and Comparability of Aggregation Data

The mechanistic account given above is qualitative, and the practical value of a mechanism in this field depends on whether it can be tested against numbers. The quantities that allow this are well established for aqueous systems and can, with the qualifications set out below, be applied to IL media. Table 1 lists them together with their definitions, the methods used to obtain them, and the specific difficulties encountered when they are measured in an ionic liquid.

The critical micelle concentration is the primary threshold quantity and also enters the standard thermodynamic treatment. For a nonionic surfactant, the Gibbs energy of micellization per mole of surfactant is obtained from the CMC expressed as a mole fraction, X_CMC_, through ΔG°_mic_ = RT ln X_CMC_, and for an ionic surfactant the same relation carries a correction for the fraction of headgroup charge that is neutralized by bound counterions, ΔG°_mic_ = (1 + β) RT ln X_CMC_, where β is the counterion binding fraction [53,57]. The binding fraction is itself informative, because it reports how effectively the medium compensates the headgroup charge, and in IL media it reflects both the bulk ion concentration and the specific affinity of the prevailing ions for the headgroup. The enthalpy of micellization is measured directly by isothermal titration calorimetry, and the entropy follows from ΔS°_mic_ = (ΔH°_mic_ − ΔG°_mic_)/T. The aggregation number Nagg, obtained from time-resolved fluorescence quenching or from scattering, provides the structural counterpart to these energetic quantities and is the parameter that most directly distinguishes aggregate growth from a change in aggregate shape.

Three cautions apply when these relations are used in IL media. The phase-separation and mass-action models that underlie them assume a well-defined threshold and, for the ionic case, an ideal solution of monomers and a single bound counterion species. In an IL neither assumption holds exactly, because the counterion reservoir is the solvent itself and the monomer is solvated by an anisotropic ionic medium [36,94]. Second, the numerical values of ΔH°_mic_ in IL media are frequently small in magnitude, so that the entropic term dominates and the enthalpy of micellization measured in water cannot be used as a guide to its value in an IL. Third, because ΔG°_mic_ depends on the logarithm of the CMC, an error of a factor of two in the CMC shifts it by only about 1.7 kJ mol^−1^ at room temperature; differences in this size should not be interpreted mechanistically unless the measurement conditions are matched.

Table 4 collects representative systems from the five classes defined in Section 1.2 and states, for each, the ionic liquid composition, the surfactant class, the water content, the temperature, the aggregation outcome, the technique used to resolve it, and the reference. It is intended to be read as an evidence map rather than as a league table, because the entries differ in the strength of the structural evidence behind them and because several of the studies do not report the water content or the equilibration time.

Three patterns emerge when the entries are read together. First, the CMC of a given surfactant falls as the length of the cation alkyl chain of the IL increases, and this trend is observed in both protic and aprotic media, which is consistent with a solvophobic contribution that is strengthened as the non-polar volume of the solvent grows [42,86,87]. Second, the anion exerts a control that is not captured by its size or charge alone. The direction of the effect depends on whether the anion competes with the headgroup for solvation or promotes ion-pair separation, so that anions of similar size can shift the CMC in opposite directions [43,49,91]. Third, the transition from spherical to elongated or lamellar structures is most often reported in systems in which the headgroup area is reduced, either by counterion binding or by aromatic stacking between headgroups, and least often in systems in which the headgroup is strongly solvated by a protic network [97,118].

The comparability of these comparisons is limited by three inconsistencies in the primary literature. Water content is reported in a minority of the studies, although it is known to change the CMC by more than the differences being discussed (Section 2.5). The method used to locate the aggregation threshold differs between studies, and fluorescence and surface-tension methods commonly give thresholds that differ by tens of per cent even for the same system in water [120]. And the temperature at which the measurement was performed is sometimes stated only as ambient. These limitations, rather than an absence of measurements, are the principal obstacle to the quantitative structure–property relationships that the field aims to establish, and the reporting practices that would remove them are set out in Section 5.7.

## 4. Structural Types of Surfactants in Ionic Liquids and Characterization Methods

### 4.1. Types of Self-Assembled Structures

Having established the mechanistic framework of surfactant self-assembly in ILs (Section 3), we now turn to the structural manifestations of these mechanisms [44,121]. The following sections describe the morphological characteristics of each aggregate type—micelles, reverse micelles, lyotropic liquid crystals, microemulsions, and worm-like micelles—and the experimental methods used to resolve them. While the formation of each structure is governed by the driving forces discussed in Section 3, here we focus on their geometric features, phase boundaries, and characterization challenges rather than revisiting the underlying thermodynamics.

The five architectures described below are not arbitrary categories; each occupies a region of a common space defined by the effective packing geometry, the strength of the solvophobic driving force, and the ability of the medium to screen or bridge the headgroup charge. Table 7 maps that space, and it should be read as a companion to the mechanistic framework of Section 3 rather than as a set of independent classifications. Three features of the map are worth noting at the outset. The boundaries between the regions are not sharp, and continuous transitions between adjacent structures, such as the growth of spherical micelles into worm-like micelles or the closing of a lamellar phase into a bicontinuous cubic one, are commonly observed. The position of a given system on the map depends on composition as much as on molecular structure, so that the same surfactant can occupy different regions in different ILs. And several regions of the map are supported mainly by scattering or microscopy data, whereas others rest on rheology or on indirect probes; the confidence attached to each entry therefore differs, and Table 8 gives the resolution limits of the techniques involved.

#### 4.1.1. Micelles and Mixed Micelles

The thermodynamic nucleation and local dynamics of micellar aggregates exhibit distinct environment-dependent control laws when moving from mixed aqueous–IL domains to neat, water-free ionic liquid matrices. In mixed aqueous–IL systems, where water remains the dominant continuous phase, the introduced IL operates primarily as an amphiphilic cosolvent or building block [41]. Here, micellization is governed by the classic, highly cooperative hydrophobic effect. As monitored by microenvironmental fluorescence sensing (e.g., Pyrene or THP-T1 probes), exceeding the CMC triggers a sharp, cooperative transition that expels water molecules and packs the surfactant tails into a highly dynamic, liquid-like hydrophobic core, yielding distinct spectral intensity shifts [119]. In sharp contrast, inside a pure, water-free ionic liquid matrix, the total absence of water’s three-dimensional hydrogen-bonded network forces a complete paradigm shift in the driving force. Rather than relying on water-phobic displacement, the aggregation is initiated through localized solvophobic sorting, where the non-polar surfactant tails are forced to partition into the intrinsic non-polar nanodomains of the bulk IL to minimize disruptive contact with the cohesive polar ionic networks [97]. Because this non-aqueous sorting is inherently less cooperative, neat IL systems typically manifest significantly higher CMCs and require tighter structural matching between the surfactant architecture and the solvent matrix to overcome the nucleation barrier. Furthermore, as unveiled by time-resolved fluorescence decay and specific anion-induced quenching, the local dynamics within these neat IL clusters are remarkably suppressed. In neat ILs, micelles typically exhibit aggregation numbers (Nagg) of 20–50, smaller than in aqueous systems (where Nagg often exceeds 80 for similar surfactants), as determined by time-resolved fluorescence quenching. The micellar core is more compact and less hydrated, with restricted internal dynamics due to the high-viscosity IL matrix. This packing behavior is consistent with the electrostatic screening effects described in Section 3.3.

#### 4.1.2. Reverse Micelles

In systems where an ionic liquid serves as the continuous phase, the presence of a small amount of polar additive (such as water) may lead to the formation of reverse Micelle structures. In this case, the polar components are encapsulated within the aggregates, while the hydrophobic chains face the external ionic liquid environment [50]. Compared with conventional organic solvent systems, the nanoscale compartmentalization within the ionic liquid may exert a templating effect on the size and stability of the reverse micellar structures, thereby modulating the polarity of the internal microenvironment. Abe et al. [60] utilized small-angle/wide-angle neutron scattering (SWANS) to investigate a mixture of long-chain imidazolium ionic liquids and heavy water (D_2_O). They found that the inherent structural heterogeneity of the ionic liquid produces a significant nanoscale partitioning templating effect, enabling precise control over the geometric topology of the aggregates and the polarity of the confined water microenvironment within them by inducing the formation of complex phases such as nanoscale water pockets and double-helix cubic phases. Such structures hold potential for application in catalytic reactions and the synthesis of nanomaterials.

#### 4.1.3. Lyotropic Liquid Crystal (LLC) Phases

At elevated concentrations, surfactants can self-assemble into various lyotropic liquid crystal (LLC) phases within ionic liquid (IL) media, including lamellar, hexagonal, or cubic architectures. To eliminate the severe structural disruptions caused by thermal evaporation characteristic of traditional aqueous phases, current research prioritizes the development of lyotropic liquid crystals entirely within pure, water-free IL continuous matrices [124]. The specific LLC phase depends on surfactant concentration, temperature, and IL structure. Unlike aqueous LLCs, anhydrous IL-based systems exhibit phase stability over wider temperature ranges (typically 20–150 °C) due to the negligible vapor pressure of ILs.

The universal physical principle governing this ionic structural tuning can be fundamentally understood by examining the localized geometric parameters of the liquid crystal lattice. The self-assembly pathway driving surfactants from single micelles toward extended lamellar liquid crystal (LLC) phases relies heavily on regulating the interfacial curvature of the amphiphilic bilayer. Although originally resolved by Chen et al. [45] using small-angle X-ray scattering (SAXS) within an aqueous cosurfactant framework, the repeat distance, surfactant bilayer thickness, and IL domain segregation offer a highly transferable physical model for anhydrous systems.

In a pure, water-free IL continuous phase, this steric regulation becomes even more direct. When surfactants are ordered within neat imidazolium or protonic ILs, lengthening the cationic alkyl chain or tuning the hydrogen-bonding affinity of the solvent matrix directly modulates the hydrophobic volume, mimicking the structural transitions validated by Chen et al. [45] but completely bypassing the collaborative action of molecular water. Consequently, by adapting these established geometric models to entirely anhydrous environments, pure ionic liquid solvents can be exploited as independent, thermally stable “structural tuning media” to achieve predictable and robust macroscale liquid crystalline mesophases under extreme, non-aqueous conditions.

#### 4.1.4. Microemulsion Systems

Microemulsions are thermodynamically stable dispersions stabilized by surfactants, typically comprising two immiscible liquids [125]. In ionic liquid systems, ionic liquid-in-oil (IL/O) [126], oil-in-ionic liquid (O/IL) [127] or bicontinuous structures [123] can be formed. The polarity and viscosity of ionic liquids significantly influence the formation range and structural stability of microemulsions. Compared to conventional systems, IL-based microemulsions exhibit higher stability under extreme conditions: bar charts strongly demonstrate that, compared to conventional phosphate-buffered saline (PBS) systems, the introduction of specific biocompatible ILs (such as [Cho][Nic]) can significantly improve the thermal stability and immunogenicity of the internal encapsulants, although the viscosity of the system may limit its kinetic processes. Rahman et al. [101] performed quantitative characterization of ionic liquid microemulsions containing [C4mim][BF_4_] using depolarized dynamic light scattering (DDLS) and viscosity measurements. It was found that the interval distribution of the microemulsion phase diagram was strongly correlated with changes in the macroscopic viscosity of the system; simultaneously, due to the caging effect induced by the high-viscosity background, the system exhibited a slow diffusion mode with a delay time of up to three to four orders of magnitude, directly confirming that the high-viscosity characteristics of the system impose significant physical constraints on the dynamic processes of its internal nanodroplets.

#### 4.1.5. Worm-like Micelles and Viscoelastic Networks

Under specific ionic architectures and concentrations, surfactants in IL systems can form worm-like micelles (WLMs), thereby constructing dynamic viscoelastic network structures. Worm-like micelles (WLMs) are flexible, elongated aggregates with contour lengths exceeding 100 nm and diameters of 2–5 nm. In IL media, WLMs can form dynamic viscoelastic networks that exhibit shear-thinning behavior, as demonstrated by Yang et al. [122] in a [BMIM][AlCl_4_]-based system, where the plateau modulus (G°_N_) increased five-fold upon WLM formation [128]. Such structures hold potential applications in fields such as rheological modulation, interfacial engineering and enhanced oil recovery (EOR). This morphology is consistent with the packing-parameter regime of 1/3 < *P* < 1/2 discussed in Section 3.5.

### 4.2. Characterization Methods

Because the ionic liquid systems of interest are highly viscous and internally structured, their structural characterization requires a combination of techniques rather than a single measurement. Compared with conventional aqueous solutions, the physical properties of IL systems impose additional constraints on each of the methods described below. Table 8 summarizes the complementary capabilities of these techniques and states, for each one, the length scale it resolves, the concentration range over which it remains informative in an IL medium, the artifacts most likely to be encountered, and the cross-checks that are advisable.

Two general conclusions follow from the table. No single technique determines both the aggregation threshold and the aggregate morphology in an IL system with acceptable confidence, so a threshold obtained by one method should be confirmed by a second method with a different physical basis. In addition, the techniques that perform best at low surfactant concentration, namely fluorescence spectroscopy and surface tension, are precisely those that are most affected by the optical and interfacial properties of the IL, so calibration against a reference system is required.

#### 4.2.1. Surface Tension and Conductivity Measurements

Surface tension measurements are traditional ex situ methods used to determine the critical micelle concentration (CMC) and interfacial adsorption behavior [129]. However, inside neat ionic liquid systems, because the surface tension of the bulk solvent itself is inherently low (typically 25–35 mN/m), the magnitude of the tensiometric drop following surfactant addition is far smaller than that in conventional aqueous environments, which may limit the dynamic range of measurements and demand exceptionally high instrument sensitivity.

For conductivity measurements, while this technique is widely celebrated in aqueous chemistry for tracking the CMC via the inflection point on a concentration-conductivity curve, its utility becomes critically compromised in pure or bulk ionic liquid media. Because the continuous phase of an IL is composed entirely of organic ions, it possesses an extremely high background ionic strength. Consequently, the minute rearrangement of charge carriers or changes in the degree of counterion binding triggered by surfactant micellization may be difficult to detect, especially at the inflection point. Relying solely on conductivity can easily lead to false or less reliable interpretations in water-free IL systems. Therefore, data derived from electrochemical transport properties must be interpreted with extreme caution, and it is strictly recommended to cross-validate any aggregation thresholds using more sensitive alternative techniques, such as fluorescence spectroscopy or small-angle scattering methods.

#### 4.2.2. Spectroscopic Techniques

Ultraviolet–visible (UV–Vis) spectroscopy and fluorescence spectroscopy serve as highly sensitive tools to detect shifts in microenvironmental polarity and characterize vital intermolecular interactions during surfactant aggregation. By tracking the solvatochromic shifts in the emission peaks or monitoring the intensity ratios of specific pyrene vibronic bands, researchers can effectively pinpoint the critical micelle concentration (CMC) and infer the internal core density of the aggregates [130]. However, from a critical methodological standpoint, the implementation of these steady-state optical probes inside neat ionic liquid (IL) environments faces distinct photophysical interferences. Because common aprotic ionic liquids feature aromatic structural units such as imidazolium rings, conventional hydrophobic probes frequently engage in competitive non-covalent interactions, including aromatic π-π stacking and local hydrogen-bonding with the bulk solvent matrix itself [131]. This localized probe–solvent coupling can artificially mask the true micellar microenvironment, introducing notable background artifacts that demand meticulous blank calibration. To probe deeper ground-state interactions, Fourier transform infrared spectroscopy (FTIR) [132] and nuclear magnetic resonance (NMR) [133] spectroscopy are deployed to analyze specific variations in hydrogen-bonding networks and segment-level binding modes. Chemical shift variations in NMR are particularly powerful for distinguishing the counterion penetration depth within the aggregate shell. Nevertheless, a major physical challenge in IL-based NMR spectroscopy stems from the high bulk viscosity of the ionic continuous phase. Elevated viscosity drastically slows down molecular tumbling rates, causing shortened spin–spin relaxation times and severe spectral line broadening that compromises resolution. Consequently, high-resolution structural elucidation mandates the deployment of temperature-programmed NMR experiments or specific isotopic deuteration of the bulk IL phase to successfully resolve the heavily overlapped chemical shifts in the packed amphiphilic chains.

#### 4.2.3. Scattering Techniques

Small-angle X-ray scattering (SAXS) and small-angle neutron scattering (SANS) are powerful and widely used tools for studying the macroscopic morphology of surfactant aggregates, providing precise structural parameters such as the aggregation number, core radius, and shell thickness [134]. Despite their high precision, the methodological trade-offs between these two scattering techniques become exceptionally pronounced under the constraints of a dense, solvent-free ionic liquid matrix. For SAXS characterization, because common IL anions are heavily enriched with electron-dense heavy atoms, such as bromine, iodine, or fluorine clusters in bis(trifluoromethylsulfonyl)imide ([NTf2]^−^), the electron density contrast between the bulk IL continuous phase and the hydrocarbon core of the surfactant aggregates is severely diminished. This inherently poor contrast alignment frequently results in weak scattering intensities and ambiguous shape fitting, making ordinary ex situ SAXS insufficient for fine structural resolution. Conversely, SANS offers an unparalleled advantage in IL media; by selectively utilizing deuterated ionic liquids or executing tailored isotopic labeling of the surfactant segments, researchers can artificially manipulate the scattering length densities to completely highlight or eliminate the scattering background of specific domains, thereby offering unique structural clarity without contrast bottlenecks [135]. For tracking particle size distributions and temporal growth trends, dynamic light scattering (DLS) is universally employed [136]. However, in neat IL environments, ordinary DLS measurements can be constrained by the caging effect of the highly viscous ionic network [137]. This frictional resistance severely decelerates Brownian motion, restricting free particle diffusion and yielding artificially inflated hydrodynamic radii if evaluated through standard aqueous frameworks. Therefore, when using the Stokes–Einstein equation, the temperature-dependent viscosity value of the specific IL system must be precisely measured and input. To achieve practical guidance and differentiate true micellar diffusion from the slow collective motions of the structured IL background, the integration of depolarized dynamic light scattering (DDLS) is highly required to decouple translational diffusion from bulk transport modes.

#### 4.2.4. Molecular Simulation and Theoretical Calculations

In recent years, molecular dynamics (MD) simulations have emerged as an indispensable toolkit for decoding the nanoscale morphology and intricate intermolecular interaction networks within ionic liquid (IL) systems [138]. Advanced atomistic simulations offer deep molecular-level insights into specific driving forces, effectively unveiling the localized solvation modes of hydrophobic tails [139], the spatial distribution and topological fluctuations of hydrogen-bonded networks [140], and the long-range electrostatic screening effects inherent to ionic matrices [141].

A central challenge when MD simulations are applied to IL-based formulations is the accuracy of the force field. Conventional non-polarizable force fields frequently fail to capture the electronic polarization and charge-transfer effects that prevail in highly coordinated ionic domains, and they typically overestimate the viscosity of the simulated medium and underestimate the mobility of its ions and of the amphiphile [142]. Three remedies are in common use, each with a cost. Charge scaling, in which the formal charges of the ions are reduced by a factor usually between about 0.7 and 0.9 so as to mimic the effect of polarization and charge transfer, improves the description of transport properties and structural correlations but shifts the balance between Coulombic and dispersive interactions and therefore requires reparameterization of the dispersive terms [11,142]. Explicitly polarizable and Drude-oscillator force fields reproduce induction effects and the dielectric response more faithfully, but they are computationally expensive and comparatively few parameter sets have been validated for ILs. United-atom and coarse-grained models extend the accessible time and length scales by one to several orders of magnitude, at the price of losing the specific ion and headgroup interactions that determine the morphology in the first place [82].

Simulation design choices matter as much as the force field. Aggregate dimensions in these systems range from nanometres to tens of nanometres, and the formation of a well-defined aggregate from a dispersed initial configuration generally requires simulation times of at least several tens of nanoseconds and often substantially longer, particularly in high-viscosity ILs, where diffusion coefficients are one to two orders of magnitude smaller than in water [86]. Systems of a few thousand atoms are sufficient to describe the local solvation and hydrogen-bond structure of a single amphiphile but not to establish an equilibrium aggregate shape; system sizes of tens of thousands of atoms are needed for that purpose, and finite-size effects should be checked explicitly by comparing simulations performed in at least two box sizes. Enhanced-sampling methods, including umbrella sampling, metadynamics, and replica exchange, are required to obtain free energies of aggregation or of ion penetration, because these events occur on timescales that are inaccessible to brute-force MD [57,58].

The value of a simulation in this field therefore depends less on the sophistication of the model than on the validation strategy adopted. Simulations should be validated against at least one experimental observable that is sensitive to the same structural feature, such as the aggregate size and shape from SAXS or SANS, the aggregation number from time-resolved fluorescence quenching, or the enthalpy of micellization from isothermal titration calorimetry. Agreement with a single averaged quantity such as the CMC is not sufficient, because it can be reproduced by models that produce different aggregate structures. Simulation reports would also be more useful if they stated the force field and the charge-scaling factor, the system size, the simulation length, the number of independent replicas, and the convergence criteria applied.

## 5. Advances in Applications of Surfactant–Ionic Liquid Self-Assembly Systems

The self-assembly behavior of surfactants in ionic liquids is not only of theoretical significance at the fundamental research level but also demonstrates unique application potential in fields such as materials science [143], energy conversion [144], biomedicine [145] and separation engineering [146]. Compared with traditional molecular solvents, pristine ionic liquids provide an anhydrous continuous medium characterized by exceptional ionic strength [98], widely tunable polarities, and inherent structural designability [147]. These solvent properties enable the resulting self-assembled soft materials to exhibit greater structural diversity and higher thermal stability [148].

The discussion below is organized around structure–property–performance relationships rather than around a catalog of examples. For each application area, the self-assembled structure is identified, the property it is expected to control is stated, the performance metric that would demonstrate the effect is named, and the strength of the current evidence is assessed. Table 2 summarizes this assessment, and the sections that follow give the supporting detail and the specific qualifications that apply to each area.

### 5.1. Construction of Nanomaterials and Template Synthesis

Surfactant self-assembled structures can serve as soft templates for synthesizing structured nanomaterials within pure ionic liquid systems [39]. Micelles, reverse micelles, and lyotropic liquid crystal phases provide highly confined, non-aqueous localized environments for the controlled nucleation and directional growth of nanoparticles, nanowires, and mesoporous matrices [149]. In pure ionic liquid environments, the template effect offers distinct advantages: high thermal stability facilitates high-temperature crystalline reactions; negligible vapor pressure prevents solvent evaporation; and nanoscale compartmentalized structures modulate particle morphology [40]. By precisely adjusting the anion–cation structure of the continuous ionic liquid phase, the structural packing parameters and interfacial curvature of the surfactant aggregates can be tailored, thereby influencing the final pore size and morphology of the synthesized products [150]. The relationship between the two is nevertheless indirect: the IL composition sets the phase geometry of the template, and the template geometry sets the accessible pore size, so a change in IL composition affects the product only through an intermediate structural variable that should be measured rather than assumed.

Zhai et al., for example, achieved precise control over the internal structure of monoolein nanoparticles by introducing 13 protic ionic liquids (PILs) as modulating media [151]. Their study confirmed that altering the cation–anion pairings of the PILs induced a continuous hexagonal–cubic–lamellar phase transition, expanding the potential internal solvent nanochannels by up to 40%. This result provides evidence for the tailored design of pore sizes in macromolecular encapsulation materials. Compared with traditional solvent systems, IL-mediated templates exhibit superior structural integrity over a broader temperature range, enabling the synthesis of functionally active materials with refined morphologies [152]. Although IL-based templates offer advantages in structural stability, the high viscosity of pure ILs may restrict the diffusion kinetics of inorganic precursors into the template interior, occasionally leading to incomplete template filling [153]. A comparison with a conventional solvent-template route, in which the template phase is measured by scattering and the product structure by the same method, would allow this viscosity penalty to be quantified rather than inferred.

### 5.2. Energy Storage and Electrochemical Systems

Neat ionic liquids possess a wide electrochemical window and excellent thermal stability, making them premier candidates for electrochemical applications; consequently, integrating them with self-assembled surfactant architectures provides a robust framework for designing advanced energy devices [154]. Unlike traditional hybrid formulations that suffer from structural instability under extreme conditions, self-assembled architectures within pure, anhydrous IL electrolytes construct highly ordered ion transport pathways. This modulates electrode–electrolyte interfacial interactions [155], thereby effectively suppressing lithium/zinc dendritic growth and enhancing interfacial stability [156].

For instance, lyotropic liquid crystal structures can form regular ion-conducting networks [157]. leveraging the bulk electrostatic shielding effect (discussed in Section 3.3). The abundant organic ion pairs in pure ILs suppress long-range electrical double-layer repulsion at the electrode surface, broadening the operational voltage window. Furthermore, continuous worm-like micelles construct uninterrupted ion transport pathways to enhance overall ionic conductivity [158]. Additionally, the improved interfacial wettability of ionic liquids towards nanostructured carbon electrode materials helps to reduce interfacial impedance [159] and optimize cycling stability under high-temperature and high-voltage conditions [160].

Machine-learning-assisted design of these systems is treated separately in Section 5.6, where the available descriptors, the current limitations of the underlying data, and the realistic opportunities for predictive modeling are assessed.

Claims that self-assembled surfactant structures improve electrochemical performance should be judged against device-level metrics, and the present literature does not always support the strong form of these claims. Three requirements follow. First, the performance metric should be reported for a complete cell rather than for a half-cell or for the electrolyte alone, and it should include the quantity that the claimed mechanism is expected to change. For lithium or zinc metal anodes, these quantities are the critical current density at which dendrite penetration occurs, the Coulombic efficiency over a stated number of cycles, and the areal capacity; for supercapacitors, they are the capacitance retention and the equivalent series resistance before and after cycling. For any device, the rate capability, the measurement temperature, and the cycle number reached should be stated [154,156,160]. Second, the comparison should be made against a formulation that differs only in the presence or the amount of the surfactant or of the self-assembled structure, so that the contribution of the structure can be separated from the contributions of the ionic liquid, the salt, and the separator. Third, the link between the structure and the performance should be demonstrated rather than asserted: the presence and the dimensions of the aggregates under operating conditions should be established by scattering, rheology, or in situ spectroscopy, and any structural change attributed to cycling should be measured. Where these requirements are not met, the improvement is described as correlative. Table 2 records the current evidence status for each application area.

### 5.3. Biomedical and Drug Delivery Systems

Nanostructured drug delivery systems, including liposomes, polymeric micelles, nanoemulsions, solid lipid nanoparticles, nanostructured lipid carriers, and lyotropic liquid crystalline nanoparticles, have been extensively explored to improve drug solubility, stability, bioavailability, and targeting [161]. In the biomedical field, ionic liquid-based self-assembled systems can serve as potential drug carriers, being particularly suitable for the encapsulation and controlled release of poorly water-soluble, hydrophobic drugs [162]. Anhydrous micelle and microemulsion structures provide a hydrophobic core environment for drug molecules, enhancing their solubility and stability [163]. The structural tunability of ionic liquids enables them to alter aggregate core polarity, optimize drug–carrier thermodynamic interactions, and modulate release rates [164]. Furthermore, functionalized surface-active ionic liquids can introduce specific recognition groups onto the surface of self-assembled structures, thereby enabling active targeted delivery [165]. Certain responsive ionic liquids undergo structural changes in response to temperature, pH or enzymatic action, causing the aggregates to dissociate or reorganize, thereby enabling stimulus-responsive release [166].

Despite this promising therapeutic potential, the intrinsic cytotoxicity and poor biodegradability of traditional imidazolium-based ILs have long presented severe biosafety bottlenecks. To address this challenge, recent biomedical breakthroughs have witnessed a comprehensive transition toward fully biocompatible, bio-derived protic ionic liquids (PILs), specifically utilizing choline- and amino acid-based formulations. Advanced drug delivery architectures now successfully leverage the unique, dynamic hydrogen-bonding networks of these green ILs to spontaneously encapsulate sensitive biopolymers or labile hydrophobic therapeutics. These biocompatible IL–surfactant assemblies not only drastically enhance the transdermal and cellular permeability of low-solubility drugs via non-disruptive lipid bilayer fluidization, but also exhibit smart, multi-stimuli-responsive properties (such as temperature- or shear-induced gelation–sol transitions) that enable targeted localized drug release.

Biomedical claims in this area rest on a different kind of evidence than that used in the materials and energy sections, and the distinction between demonstrated biocompatibility and proposed biomedical potential should be made explicitly. Four issues require attention, and they are the reason why the evidence status recorded in Table 2 for drug delivery is cautious.

Cytotoxicity. The toxicity of an IL-based formulation is determined by the IL, by the surfactant, and by their combination, and not by the IL alone [18,37]. Imidazolium cations with longer alkyl chains are generally more cytotoxic than their short-chain counterparts, and the anion contributes through its lipophilicity and its susceptibility to hydrolysis; the mixture can be more toxic than either component, because the surfactant increases the membrane partitioning of the IL. Toxicity values should therefore be reported for the complete formulation, with the cell line, exposure time, assay, and concentration range stated. Values obtained for an IL in water at concentrations far from those used in a self-assembled formulation should not be presented as evidence of safety.

Biodegradability. The biodegradable fraction of a formulation depends on the surfactant as well as on the IL. Bio-derived cations such as choline and amino-acid-derived anions are more readily degraded than imidazolium or quaternary ammonium cations, and the presence of a fluorinated anion generally precludes biodegradation [147]. Degradation data should be reported for the formulation rather than inferred from the cation class.

Pharmacokinetics. Encapsulation changes the distribution of the drug, and the fate of the carrier must be assessed separately from that of the payload. The quantities that determine whether a carrier is viable, namely the circulation half-life, the tissue distribution, the clearance route, and the release kinetics of the encapsulated drug in a physiologically relevant medium, are rarely reported for IL–surfactant assemblies [38,162]. Reports that give only the encapsulation efficiency and an in vitro release profile do not establish a pharmacokinetic advantage.

Residual ionic liquid toxicity and clearance. When a carrier is administered in vivo, the IL released on disassembly is a solute with its own distribution and clearance, and its accumulation must be considered. This is particularly important for formulations in which the IL is present in large excess relative to the drug.

The practical conclusion is that the biomedical potential of these systems is well motivated but not yet demonstrated at the level expected for pharmaceutical development. The most useful recent work has moved towards bio-derived ILs with characterized degradation products [18,145], and progress would be accelerated if the four items above were reported routinely.

### 5.4. Enhanced Oil Recovery and Interfacial Modulation Engineering

Under high-salinity and high-temperature reservoir conditions, conventional surfactants are prone to precipitation or degradation, limiting their application [167]. The introduction of ionic liquids can significantly improve the system’s salt tolerance and thermal stability [168]. Consequently, IL–surfactant mixtures typically exhibit reduced interfacial tension (IFT), enhanced emulsion stability, and superior wettability control [46]. The electrostatic shielding effect in high-ionic-strength environments helps to reduce headgroup repulsion, thereby enhancing interfacial adsorption capacity [169]. Some ionic liquids can also alter rock surface wettability, improving oil displacement efficiency [170]. To facilitate large-scale industrial deployment, recent advances in enhanced oil recovery focus on the precise tuning of WLM viscoelastic networks by exploiting ion-specific solvation in anhydrous IL media. Balancing electrostatic interactions and solvophobic packing parameters has enabled the development of ultra-high-temperature hydraulic fracturing fluids. These self-assembled fluid networks retain their structured macroscopic viscosity under severe reservoir shear and thermal stresses yet can be efficiently broken down into low-viscosity monomers upon chemical triggering without leaving damaging carbon residues in formation pores, thereby providing a sustainable alternative for deep geological energy extraction.

The evidence for these effects is strongest at the formulation level, where interfacial tension, emulsion stability, and wettability are measured directly, and weaker for the attribution of the improvement to a specific self-assembled structure. Where a claim is made that a worm-like micelle network rather than a molecularly dispersed surfactant is responsible for the rheological behavior, the network should be characterized by scattering or by rheology in a range where the two hypothesized states differ, and the temperature and salinity at which the network persists should be compared against the operating conditions of the reservoir.

### 5.5. Green Separation and Extraction Technologies

Whether in hybrid continuous domains or in pure extraction phases, ionic liquid-based self-assembled systems offer unique advantages for liquid–liquid extraction [171] and gas separation [172]. In liquid–liquid extraction, self-assembled architectures drive the separation of target molecules through selective solubilization and interfacial enrichment, and tailoring the ionic liquid structure directly optimizes the microenvironment polarity and the separation selectivity [173]. For gas separation, particularly CO_2_ capture, surfactant incorporation improves the dispersion of the ionic liquid within membrane materials, expanding the gas–solvent contact area and improving the mass-transfer efficiency [174,175,176].

The low volatility and tunable polarity of these systems are frequently presented as evidence of superiority over traditional organic solvents, but the comparison is not meaningful without a functional unit and a system boundary, and the high viscosity of the medium may limit mass-transfer rates in practice [177]. The efficacy of surfactant–ionic liquid systems depends on tailoring self-assembly pathways so as to control the structure–property relationship deliberately, and future adoption depends on mitigating viscosity bottlenecks, reducing cost, and establishing biosafety.

The description of ionic liquids as green solvents is a claim about a life cycle rather than about a set of physical properties, and the low volatility that originally motivated the label does not by itself establish a favorable environmental profile. Four factors qualify that description, and they should be considered together [37,147].

Synthesis burden. Most ILs are not obtained directly from renewable feedstocks. Their preparation typically requires alkylation, anion metathesis, and purification, and the associated energy consumption, solvent use, and waste generation are rarely reported alongside the application result. The preparation of a fluorinated anion such as bis(trifluoromethylsulfonyl)imide in particular carries a substantial synthesis and waste burden.

Purification and impurities. Residual halide, unreacted amine, and water are common in as-prepared ILs and affect both the physical properties and the toxicity of the material [88]. Application studies frequently do not report the purity of the IL used, which makes both the reproducibility and the environmental assessment incomplete.

Persistence and ecotoxicity. Low volatility reduces atmospheric emission but also removes the principal abiotic degradation pathway available to volatile organic solvents, so an IL that is resistant to hydrolysis and to biodegradation can persist in water and soil. Measured ecotoxicological endpoints for ILs and for IL–surfactant mixtures remain sparse relative to the number of ILs in use, and structure–toxicity relationships are available only for the most common cation families [18,37].

Recyclability and recovery. IL-based separation processes are frequently described as recyclable on the basis of a small number of reuse cycles, without reporting the recovery yield per cycle, the accumulation of impurities, or the energy required for regeneration. A recovery step that requires distillation, membrane processing, or solvent extraction can dominate the life-cycle impact of the process.

A defensible claim about sustainability should therefore be made in comparative and quantitative terms: relative to which existing process, on the basis of which functional unit, and with which assumptions about IL recovery and end-of-life. Life-cycle assessment data for IL–surfactant systems remain scarce, and this is one of the more consequential gaps identified in this review (Section 5.7).

Refs. [174,175,176]. Compared with traditional organic solvents, this system offers advantages in terms of safety and sustainability; however, its high viscosity may limit mass transfer rates [177]. Ultimately, the efficacy of surfactant–ionic liquid systems relies on tailoring self-assembly pathways to precisely control structure–property relationships. Future widespread adoption depends on mitigating viscosity bottlenecks, reducing costs, and ensuring biosafety.

### 5.6. Machine-Learning-Assisted Design of Ionic Liquid–Surfactant Systems

Machine learning is beginning to be applied to the prediction of surfactant and IL properties, and it is worth being precise about where it can help. The relevant task in this field is not the prediction of an isolated property but the prediction of an assembly outcome: given an IL cation, an IL anion, an amphiphile, a composition, and a temperature, what are the CMC, the aggregate morphology, and the dimensions of the aggregate? Each of these outputs depends on the other components of the mixture and on the water content, so the learning problem is a structure–property mapping over a high-dimensional and sparsely sampled composition space.

Descriptors. The descriptors that have proved useful for the component properties fall into four groups. The first are constitutional and topological descriptors of the cation and anion, including alkyl-chain length and branching, ring type, and symmetry. The second are quantum-chemical and solvation descriptors, of which the COSMO-RS sigma profile and the associated surface-charge moments are the most transferable, because they encode the polar and non-polar character of a molecule without requiring an experimental measurement [88]. The third are physical-property descriptors such as molar volume, viscosity, and dielectric constant, which must themselves be predicted if they are not measured. The fourth are process descriptors, in particular temperature, water content, and the molar ratio of surfactant to IL, which are as influential as molecular structure and are frequently omitted. For the amphiphile, chain length, headgroup charge, counterion identity, and the presence of aromatic or fluorinated groups are the descriptors with the clearest mechanistic link to the packing parameter.

Target properties and data availability. The largest obstacle is the data. Published CMC values for IL-containing systems number in the hundreds to low thousands, are distributed unevenly across system classes, and are measured under conditions that differ in water content, temperature, and method [118]. A model trained on such a compilation learns the measurement conditions as much as the chemistry, and its apparent accuracy does not transfer to a new system. The same limitation applies with greater force to aggregate morphology, where the label is categorical and the classes are imbalanced, and to device-level performance, where the number of reported data points is very small. Curated databases and agreed reporting standards would do more for predictive modeling in this field than additional model complexity.

Validation. Reported metrics should be based on splits that prevent information leakage between chemically similar compounds, for example scaffold or cluster splits rather than random splits, and should include an external test set of systems that were not used in fitting. Where a model predicts a continuous quantity such as the CMC, the prediction should be accompanied by an uncertainty estimate, and accuracy should be reported in the units in which the quantity is used, that is, on a logarithmic scale for the CMC. For IL systems, accuracy should be reported separately for protic and aprotic media and for neat and water-containing systems, because the underlying physics differs between them (Section 2.4).

Realistic opportunities. Three applications are within reach in the near term. The first is the prediction of the CMC of conventional surfactants in a specified IL or IL–water mixture, using descriptors of the medium combined with the surfactant structure; existing graph-based models for the temperature dependence of CMCs in water indicate the accuracy that is attainable and the data requirements involved [67,68]. The second is the screening of ILs for a target property, such as low viscosity at a given water content or low toxicity within a cation family, where the property depends mainly on molecular structure and the available datasets are larger [88,175]. The third is the use of surrogate models to accelerate molecular simulation, for example by predicting the outcome of a simulation from a cheaper descriptor set and reserving the simulation budget for the candidates that are most informative.

What machine learning cannot yet do is predict aggregate morphology in a new IL–surfactant system with useful reliability. The training data do not exist, the relevant descriptors are not established, and the underlying problem involves collective phenomena that are not determined by molecular structure alone. Claims that computational screening can now replace experimental determination of self-assembly behavior in ILs are not supported by the available evidence, and the more defensible position is that data-driven models can prioritize experiments but not substitute for them.

### 5.7. Challenges and Future Directions

Despite rapid progress, the field has not reached the point at which the self-assembly behavior of a new IL–surfactant system can be predicted with useful accuracy or scaled to industrial practice. The obstacles are not primarily conceptual; they are obstacles of measurement, reporting, and material supply. The paragraphs below set out the specific barriers and the actions required to remove them, which are summarized in Table 9.

Reproducibility and standardized reporting. Two studies of the same nominal system frequently report different CMCs and different morphologies, and the difference can usually be traced to a variable that was not reported: the water content of the IL, its halide or amine impurity level, the purity and homolog distribution of the surfactant, the equilibration time before measurement, or the calibration of the instrument. The single most effective remedy is agreement on a minimum reporting set for this field, comprising the IL batch and its water content determined by Karl Fischer titration, the impurity levels where relevant, the surfactant purity and its CMC in a reference solvent, the temperature, the equilibration time, the method by which the aggregation threshold was determined, and the criterion used to locate it. Journals publishing work in this area could require this information as supplementary data at negligible cost to authors.

Viscosity and transport limitations. The high viscosity of most ILs slows the approach to aggregation equilibrium and complicates every dynamic measurement. Reported CMCs may be apparent rather than equilibrium values if mixing or equilibration was insufficient, and diffusion-based measurements such as dynamic light scattering must be analyzed with the temperature-dependent viscosity of the medium rather than with a water-referenced value [86,137]. Reported aggregate sizes should state the viscosity used in the analysis and the model applied.

Impurities and water sensitivity. Residual halide and water alter interfacial behavior, IL nanostructure, and toxicity, and their effects are difficult to separate retrospectively from the effects of the IL itself [98,99]. Studies that aim to establish the intrinsic behavior of a system should report and control both, and studies that use commercial ILs as received should state this explicitly.

Scale-up and cost. The applications that drive this field, particularly electrochemical devices and enhanced oil recovery, require ILs in quantities and at costs that are not currently available. The cost of a common IL is one to three orders of magnitude higher than that of the commodity solvent it would replace, and the recovery and regeneration step adds to it. Progress on scale-up therefore depends on the use of low-cost cation and anion families, on the reduction in the number of synthetic steps, and on the demonstration of closed-loop recovery at pilot scale rather than in a laboratory batch. Case studies that report the recovery yield per cycle and the quality of the recovered IL would be more informative than further demonstrations of a single-cycle application.

Toxicity and environmental fate. Structure–toxicity and structure–biodegradability relationships exist for the main cation families, but data are sparse for the anions, for bio-derived ILs, and for mixtures with surfactants [18,37]. The most useful additions would be measurements on the formulation as used, in a medium that reproduces the intended application, together with identification of the degradation products.

Quantitative predictive design. Predicting morphology and performance from molecular structure requires three things that are not yet in place: a mechanistic model validated against structural measurements rather than against a single averaged quantity, a dataset that is large enough and sufficiently standardized to support data-driven modeling, and a set of descriptors that capture the medium as well as the molecule. The construction of an open, curated database of aggregation data for IL-containing systems, with the reporting fields listed above, is the prerequisite for the remaining steps and is the highest-value investment available to the field. The overall trajectory is from a phenomenon-driven field towards a design-driven one, and the transition depends less on new instrumentation than on making the existing measurements comparable with one another.

## 6. Conclusions

This review has examined the self-assembly of surfactants in ionic liquids, from molecular driving forces through aggregate structure to application performance, and it has done so within an explicitly defined set of system classes and with explicit statement of the literature-search strategy. Four conclusions follow.

Self-assembly in IL media is governed by the same set of non-covalent interactions that operate in water, but their relative magnitudes and their ordering differ, and the differences depend on the chemical identity of the IL rather than only on its polarity. Solvophobic interactions, electrostatic screening through ion penetration, hydrogen bonding, and van der Waals and π-π stacking interactions jointly determine whether aggregation occurs, how dense the aggregate becomes, which interface is stabilized, and which morphology is selected. The frequently quoted sequence in which these contributions are said to operate as successive stages is a useful heuristic for some systems and fails predictably in others, most clearly in strongly hydrogen-bonded protic ILs, in low-ionicity ILs where screening is weak, and for nonionic and zwitterionic amphiphiles.

Quantitative comparison across the literature is at present limited by inconsistent reporting rather than by the absence of measurements. The thermodynamic and structural quantities needed to compare systems, namely the CMC, the aggregation number, the counterion binding fraction, and the enthalpy, entropy, and Gibbs energy of micellization, are obtainable by established methods, but they are reported under conditions, in particular water content and temperature, that differ between studies and are often unstated. Making these measurements comparable is a prerequisite for the quantitative structure–property relationships that the field aims to establish.

The structural and application literature is uneven in evidential strength. The formation of micelles, reverse micelles, lyotropic liquid crystals, microemulsions, and worm-like micelles in IL media is well documented where direct structural measurements have been made, and the morphology–composition-interaction map presented here provides a framework for organizing those results. In the application areas, the clearest evidence concerns the use of IL-based templates and of IL–surfactant formulations in interfacial and separation processes; the evidence linking self-assembled structures specifically to electrochemical device performance is more often correlative than causal, and the biomedical literature has largely demonstrated encapsulation and in vitro release rather than a pharmacokinetic or toxicological advantage.

The principal obstacles to progress are now practical. High viscosity, sensitivity to residual water and impurities, the cost and environmental burden of IL synthesis, and the absence of agreed reporting standards all limit both the comparability of results and the translation of the field into practice. Addressing them by curating and standardizing aggregation data, validating simulation against structural measurements, and assessing IL–surfactant formulations on a life-cycle basis would move the field from structural controllability towards functional predictability.

## Figures and Tables

**Table 1:** Quantitative parameters used to characterize surfactant self-assembly in ionic liquid media.

Parameter	Symbol	Definition and How It Is Obtained	What It Reports in an IL Medium	Principal Difficulty
Critical micelle concentration	CMC, XCMC	Threshold concentration determined by surface tension, conductivity, fluorescence, or scattering	Whether aggregation is favored and how strongly	Threshold is often broad; value depends on method and criterion used
Standard Gibbs energy of micellization	ΔG°_mic_	RT ln XCMC (nonionic); (1 + β) RT ln XCMC (ionic)	Total driving force for transfer of the amphiphile into the aggregate	Sensitive to the value of XCMC and to the model assumed
Counterion binding fraction	β	From the conductivity or potentiometric degree of dissociation	Extent of headgroup charge compensation by the medium	Difficult to separate from bulk ionic conductivity in a neat IL
Enthalpy of micellization	ΔH°_mic_	Measured directly by isothermal titration calorimetry	Balance between tail desolvation and headgroup interactions	Residual water content shifts the measured value appreciably
Entropy of micellization	ΔS°_mic_	(ΔH°_mic_ − ΔG°_mic_)/T	Solvent reorganization and chain disorder	Depends on two independently measured quantities
Aggregation number	Nagg	Time-resolved fluorescence quenching; SAXS or SANS model fitting	Size of the aggregate and degree of growth	Quencher partitioning is altered by the IL; model dependence in scattering
Aggregate dimensions	Rcore, Rh, d, d0	SAXS or SANS, dynamic light scattering, cryo-microscopy	Shape, core radius, and phase repeat distance	Contrast, viscosity correction, and polydispersity artifacts
Interfacial parameters	Γmax, Amin	Surface-tension isotherm and its analysis	Headgroup area and packing at the interface	Low surface tension of the neat IL reduces the measurable range

**Table 2:** Structure–property–performance relationships in surfactant–ionic liquid systems and the current strength of the supporting evidence.

Application Area	Self-Assembled Structure	Property It Controls	Performance Metric Affected	Evidence Status
Nanomaterial synthesis	Micelles, reverse micelles, lyotropic liquid crystals	Confinement volume and interfacial curvature	Pore size, particle morphology, phase purity	Demonstrated where phase identity and product structure are both measured
Energy storage	Lyotropic liquid crystals and worm-like micelle networks	Ion transport pathway continuity and interfacial wetting	Ionic conductivity, interfacial resistance, cycling stability, dendrite onset current density	Partly correlative; rarely resolved to the specific self-assembled structure under operating conditions
Drug delivery	Micelles and microemulsions with a hydrophobic core	Solubilization capacity and release kinetics	Encapsulation efficiency, in vitro release, transdermal flux	Demonstrated at the formulation level; pharmacokinetic and toxicological advantage largely unproven
Enhanced oil recovery	Worm-like micelle networks and interfacial films	Interfacial tension and viscoelasticity of the displacing fluid	Interfacial tension reduction, incremental oil recovery, thermal stability	Demonstrated at laboratory and core-flood scale; field-scale data absent
Separation and extraction	Micelles, microemulsions, and self-assembled membranes	Selective solubilization and transport	Distribution coefficient, selectivity, permeance, regeneration yield	Demonstrated for selected systems; life-cycle and recovery data scarce

**Table 3:** Classification of ionic liquid–amphiphile systems used in this review.

Class	Continuous Phase	Amphiphile	Dominant Driving Force	Treatment in This Review
I. Neat IL medium	IL, no deliberate cosolvent	Conventional surfactant or SAIL	Solvophobic partitioning; ion penetration	Section 2.2, Section 2.3, Section 2.4, Section 2.5, Section 3.2, Section 3.3, Section 3.4, Section 3.5, Section 3.6 and Section 4.1
II. IL–water mixture	Water-rich, IL as cosolvent or electrolyte	Conventional surfactant, often ionic	Hydrophobic effect modified by ion-specific effects	Section 3.3, Section 4.1.1 and Section 5.5
III. Surface-active ionic liquid (SAIL)	IL or IL–water, amphiphile is itself an IL	Long-chain or functionalized IL cation or anion	Self-association with strong medium matching	Section 2.3, Section 3.4, Section 3.7 and Section 4.1
IV. IL as cosurfactant	Water–oil or IL–oil	Conventional surfactant with IL in the interfacial film	Interfacial curvature and tension control	Section 3.5, Section 4.1.3 and Section 5.4
V. Conventional surfactant in IL	Neat IL or IL-rich mixture	Cationic, anionic, nonionic or zwitterionic	Solvophobic effect with specific ion effects	Section 3.2, Section 3.3, Section 3.4, Section 3.5, Section 3.6, Section 4.1, Section 5.1, Section 5.2 and Section 5.3

**Table 5:** Search protocol and eligibility criteria applied to the literature.

Item	Specification
Databases searched	Web of Science, Scopus, Google Scholar, plus backward and forward citation tracking
Search window	January 2010 to March 2026; earlier work cited only where conceptually foundational
Search string (example)	(“ionic liquid*” OR “molten salt*” OR “surface-active ionic liquid*”) AND (surfactant* OR amphiphile* OR “block copolymer*”) AND (self-assembl* OR micell* OR aggregat* OR “liquid crystal*” OR microemulsion* OR “worm-like micelle*”)
Inclusion criteria	Experimental or computational evidence on amphiphile self-assembly in an IL-containing medium; or physicochemical data (CMC, aggregation number, thermodynamic parameters, aggregate dimensions, interfacial properties) for such a system; peer-reviewed and with primary data
Exclusion criteria	IL present only as a trace additive or impurity; aqueous surfactant behavior with no IL; IL used only as a reaction medium, extractant or electrolyte without analysis of self-assembly; conference abstracts, patents, and non-peer-reviewed items without primary data
Screening	Title and abstract screening, then full-text screening
Extracted fields	IL cation and anion; surfactant identity, class, and purity; water content; temperature; aggregation threshold and method; aggregate morphology and technique; thermodynamic and structural parameters
Known limitations	Non-blind selection, no formal risk-of-bias assessment, publication bias against null results, and inconsistent reporting of water content and purity in the primary literature

**Table 4:** Representative ionic liquid-surfactant systems and the assembly data reported for them. Part A gives the system identity and composition; Part B gives the measurement conditions and the reported assembly outcome. Row numbers correspond between the two parts. n.r. indicates that the quantity is not reported in the cited study. Part B. Measurement conditions and the reported assembly outcome; row numbers correspond to Part A.

Part A					
**No.**	**Ionic Liquid (Cation/Anion)**	**Surfactant and Class**	**Water Content**	**Class**	**Reference**
1	Ethylammonium nitrate (PIL)	Sulfobetaine zwitterionic surfactant	n.r.	I, V	[48]
2	Imidazolium-based, variable anion	Anionic surfactant with nonionic surfactant	n.r.	II, V	[19]
3	Imidazolium chloride, variable chain length	Triton X-114 nonionic surfactant	n.r.	I, V	[49]
4	[C4mim][BF4]	Microemulsion formulation with surfactant monolayer	n.r.	I, IV	[50]
5	Long-chain imidazolium iodide	The IL itself (SAIL), with D_2_O added	D_2_O added	III	[51]
6	Imidazolium, aprotic	Imidazolium surfactant (SAIL)	n.r.	I, III	[52]
7	Quaternary ammonium IL	Amphiphilic gemini and trimeric ILs (SAIL)	n.r.	III	[53]
8	Zwitterionic SAIL, variable counterion	Zwitterionic surface-active IL (SAIL)	n.r.	III	[54]
9	Pyridinium-based SAIL, cation isomers	Pyridinium surface-active IL (SAIL)	n.r.	III	[55]
10	Imidazolium-based	Cationic surfactant	n.r.	I, V	[56]
11	1-Butyl-3-methylimidazolium hexafluorophosphate	Nonionic surfactant	n.r.	I, V	[57]
12	Imidazolium-based	Fluorinated surfactant	n.r.	I, V	[58]
13	Ammonium-based IL	The IL itself (SAIL)	n.r.	III	[59]
14	1-Decyl-3-methylimidazolium triflate	Conventional surfactant, oil recovery formulation	Aqueous	II, IV	[32]
15	Imidazolium IL with anionic surfactant	Catanionic surfactant mixture	n.r.	II, V	[60]
Part B					
**No.**	**Temperature**	**Aggregation Threshold**	**Aggregate Morphology**	**Characterization Method**	**Key Thermodynamic or Structural Value**
1	n.r.	CMC	Micelles and mixed aggregates	Tensiometry, conductivity	β and ΔG°_mic_ derived from the CMC
2	n.r.	CMC	Mixed micelles	Tensiometry, DFT	Interaction parameters
3	n.r.	Cloud point	Micelles with shifted cloud point	DLS	Micellar size-stability correlation
4	n.r.	Phase boundaries	Microemulsion nanodroplets	Depolarized DLS, viscosity	Diffusion delay of three to four orders of magnitude
5	n.r.	Water-driven phase formation	Nanoscale water pockets, double-helix cubic phase	SANS and WANS	Phase identity and repeat distances
6	n.r.	Aggregation threshold	Lamellar and ordered phases	SAXS, microscopy	Local charge compensation by penetrating anions
7	n.r.	Phase boundaries	Lamellar phases	SAXS	Interlamellar distance and packing density
8	n.r.	CMC	Micelles with chain-length-dependent size	Conductimetry, DLS	CMC decrease with chain length and ring stacking
9	n.r.	CMC	Micelles	Conductimetry	Cation isomerism effect on CMC
10	n.r.	CMC	Micelles	Isothermal titration calorimetry, conductivity	ΔH°_mic_ exothermic; ΔG°_mic_ and ΔS°_mic_ derived
11	n.r.	Surface adsorption and aggregation	Micelles	Surface tension	Adsorption isotherm
12	n.r.	CMC	Aggregates in IL	Tensiometry, scattering	Fluorinated chain effect on aggregation
13	n.r.	Surface activity and CMC	Micelles or aggregates	Tensiometry, conductivity	Cation and anion dependence
14	Reservoir conditions	Interfacial tension	Interfacial films	Interfacial tension measurement, core flooding	Oil recovery increment
15	n.r.	CMC	Catanionic aggregates	Tensiometry, isothermal titration calorimetry	Chain length and molar ratio effects

**Table 6:** Comparison of protic and aprotic ionic liquids and the consequences for surfactant self-assembly.

Property	Protic Ionic Liquids	Aprotic Ionic Liquids	Consequence for Self-Assembly
Exchangeable proton	Present, transferred from acid to base	Absent	Provides a competing hydrogen-bond donor at the aggregate interface in PILs
Hydrogen-bond network	Dense, dynamic, proton-mediated	Confined to cation–anion interactions	Hydrogen bonding is a primary determinant in PILs and a secondary modifier in AILs
Ionicity and ion pairing	Variable; strong specific ion interactions	Variable; ranges from strongly ion-paired to highly dissociated	Sets the strength of charge screening and the extent of counterion penetration
Nanostructure	Polar network with segregated alkyl domains	Polar and non-polar domains, strongly chain-length dependent	Controls the solvophobic driving force and the tail accommodation volume
Headgroup solvation	Directional hydrogen bonding plus electrostatics	Predominantly electrostatic, modified by dispersion	In PILs, monomer stabilization competes directly with aggregation
Dominant control of CMC	Headgroup hydrogen-bond competition	Cation alkyl-chain length and anion ion-pairing strength	Explains why the same surfactant gives different CMCs in the two families
Dominant control of morphology	Interfacial hydrogen bonding and chain segregation	Charge screening and specific ion penetration	Requires separate morphology maps for the two families
Representative systems	Ethylammonium nitrate; choline and amino-acid ILs	Imidazolium, pyrrolidinium and ammonium ILs with fluorinated or halide anions	Cautions against generalizing mechanisms across families

**Table 7:** Morphology–composition-interaction map for surfactant aggregates in ionic liquid media.

Aggregate Architecture	Packing Parameter Regime	Interaction Balance That Favors It	Compositional Signature	Typical Evidence	Example Systems
Spherical micelles	p ≤ 1/3	Weak interchain attraction; large effective headgroup area; headgroup fully solvated	Short-chain surfactant; weakly coordinating anion; protic medium that solvates the headgroup	Fluorescence quenching, DLS, SANS	[47,97,121]
Rod-like or ellipsoidal micelles	1/3 < p ≤ 1/2	Moderate chain attraction; partial charge compensation at the headgroup	Medium-chain surfactant; anion capable of ion pairing; increasing IL chain length	SANS, SAXS, rheology	[43,49]
Worm-like micelles and viscoelastic networks	1/3 < p < 1/2	Strong anisotropic chain stacking; headgroup stiffening by aromatic or π-stacking interactions	Aromatic headgroup or aromatic IL cation; concentration above the entanglement threshold	Rheology, cryo-microscopy, SANS	[122]
Lamellar and liquid crystalline phases	1/2 < p ≤ 1	Low interfacial curvature; strong headgroup charge compensation; stiff interfacial film	Double-chain or gemini surfactant; IL present as cosurfactant or at high concentration	SAXS, optical microscopy, birefringence	[45,118]
Reverse micelles and microemulsions	p > 1 or interfacial film stabilizing a polar core	Headgroup strongly solvated; polar additive available to form the core	Trace to moderate water content in a hydrophobic IL; surfactant with low interfacial tension	SANS and WANS, DDLS, phase diagrams	[50,91]
Bicontinuous microemulsions and cubic mesophases	Interfacial film near zero mean curvature	Balanced hydrophilic-lipophilic interactions; low bending rigidity	Tuned IL–water–surfactant ratio; often a narrow composition window	SAXS, SANS, conductivity mapping	[101,123]

**Table 8:** Complementary capabilities, resolution limits, and artifacts of the techniques used to characterize surfactant self-assembly in ionic liquid media.

Technique	Information Obtained	Accessible Length Scale or Resolution	Concentration Range	Principal Artifacts in IL Media	Advisable Cross-Check
Surface tension	CMC and interfacial adsorption	Not a length-resolved method; detects the threshold	Low to moderate, above the tensiometric detection limit	Low surface tension of the neat IL compresses the measurable drop; slow equilibration at high viscosity	Conductimetry or fluorescence for the same threshold
Conductivity	CMC and degree of counterion dissociation	Not length-resolved; sensitive to charge-carrier mobility	Low to moderate	High background ionic strength of the medium suppresses the inflection point	Fluorescence or scattering
UV-Vis and fluorescence spectroscopy	Microenvironment polarity and CMC	Probe-scale resolution of the local environment	Low, the most sensitive of the threshold methods	Probe partitioning into the IL and π-stacking with aromatic cations mask the micellar signal	Blank calibration against the probe in neat IL; second threshold method
FTIR and NMR spectroscopy	Hydrogen bonding and segment-level binding modes	Molecular scale; site-specific	Moderate to high	High viscosity shortens relaxation times and broadens NMR lines	Isotopic labeling; variable-temperature measurement
SAXS	Aggregate shape, core radius, phase repeat distance	About 1–100 nm; resolution limited by electron-density contrast	Moderate to high	Weak contrast between a fluorinated or heavy-atom anion and a hydrocarbon core	SANS with isotopic contrast variation
SANS	Aggregate shape, core radius, shell thickness	About 1–100 nm; contrast tunable by deuteration	Moderate to high	Requires deuteration, which changes the solvent properties slightly	Comparison with SAXS on the same system
DLS and DDLS	Hydrodynamic radius and diffusion modes	About 1 nm to several micrometers	Low to moderate	Caging by the viscous network inflates apparent radii if water-based viscosity is used	Temperature-dependent viscosity of the medium; DDLS to separate modes
Isothermal titration calorimetry	Enthalpy of micellization and binding	Not length-resolved; gives the enthalpy of the process	Moderate to high	Small enthalpies and slow thermal equilibration in viscous media	Comparison with the CMC from an independent method
MD simulation	Molecular-level structure and energetics	Atomic resolution, limited by system size and simulation time	Any, as a model system	Force-field and charge-scaling errors; insufficient sampling of slow aggregation events	Validation against at least one structural experimental observable

**Table 9:** Roadmap of the principal barriers to predictive design and practical use of surfactant–ionic liquid self-assembly.

Barrier	Current State	Required Action	Indicator of Progress
Reproducibility	The same nominal system yields different CMCs and morphologies between studies	Adopt a minimum reporting set for IL batch, water content, purity, temperature, and threshold criterion	Interlaboratory agreement within a stated tolerance for a reference system
Water content	Often unreported, although it shifts the CMC by more than the effects being compared	Report water content by Karl Fischer titration on the same batch used for the measurement	Water content reported as a standard column in aggregation studies
Viscosity and transport	Dynamic measurements analyzed with water-based or unstated viscosity	Report the temperature-dependent viscosity of the medium and the model used in the analysis	Aggregate sizes reproducible between viscometric analyzes
Impurity control	Halide and amine residues rarely quantified	Report halide and residual amine content, and state when ILs are used as received	Impurity levels reported and their effect on the CMC quantified
Scale-up	Demonstrations limited to laboratory batch quantities	Demonstrate continuous or pilot-scale preparation and closed-loop recovery	Recovery yield per cycle and recovered IL quality reported
Cost	IL cost one to three orders of magnitude above the replaced solvent	Use low-cost cation and anion families and reduce the number of synthetic steps	Cost per kilogram of IL reported at pilot scale
Toxicity and fate	Data sparse for anions, bio-derived ILs, and mixtures	Measure toxicity and biodegradation on the formulation as used	Structure-toxicity coverage extended to the main anion families
Recyclability	Reuse claimed from a small number of cycles	Report recovery yield, impurity accumulation, and regeneration energy per cycle	Life-cycle comparison against the incumbent process
Predictive morphology models	No model predicts aggregate morphology in a new system	Validate mechanistic models against structural observables, not the CMC alone	Blind prediction of morphology for a held-out system
Data infrastructure	Data dispersed, heterogeneous, and biased towards positive results	Build an open curated database with a standard field set, including null results	Public dataset with documented provenance and versioning

## Data Availability

No new data were created or analyzed in this study. Data sharing is not applicable to this article.
